# Variable Flip Angle Optimization for Fetal Brain Imaging With Reduced Specific Absorption Rate

**DOI:** 10.1002/nbm.70075

**Published:** 2025-07

**Authors:** Dominique Franson, Camilo Calixto, Hongli Fan, Camilo Jaimes, Clemente Velasco-Annis, Suely Fazio Ferraciolli, Cesar Alves, Edward Yang, Stephen Cauley, Ali Gholipour

**Affiliations:** 1 Siemens Medical Solutions USA Inc, Boston, Massachusetts, USA; 2 Department of Radiology, Boston Children’s Hospital, Boston, Massachusetts, USA; 3 Harvard Medical School, Boston, Massachusetts, USA; 4 Massachusetts General Hospital, Boston, Massachusetts, USA; 5 Department of Radiological Sciences, and Department of Electrical Engineering and Computer Science, University of California Irvine, Irvine, California, USA

**Keywords:** fetal brain, fetal MRI, HASTE, single-shot turbo spin echo, specific absorption rate, variable flip angle

## Abstract

Half-Fourier Acquisition with Single-Shot Turbo Spin Echo (HASTE) scans are routinely used for fetal brain imaging, but they have a high specific absorption rate (SAR) and can be inefficient due to SAR limits. Here, we have designed an optimized variable flip angle (VFA) pattern for fetal HASTE neuroimaging to reduce both the SAR and repetition time (TR) of the HASTE sequence while maintaining similar image quality to standard fetal HASTE imaging with a constant flip angle (CFA). The VFA pattern was optimized by minimizing the difference in expected signal between the VFA and CFA scans while constraining the SAR of the VFA scan to no more than 65% of the SAR of the CFA scan and reducing the TR by 29%. The expected signal was calculated using an extended phase graph formalism, and simulations were used to predict the performance of the two scans using different image quality metrics. The proposed VFA and standard CFA scans were tested in phantoms and fetuses at 3 T. SAR and acquisition times were recorded, and image quality was rated by three radiologists. The VFA scan showed similar signal-to-noise ratio and contrast-to-noise ratio values but slightly lower signal and relative contrast values than the CFA scan in phantom studies. In vivo, the VFA scan yielded significantly reduced SAR, measurement times, and total scan times. There was no significant difference in overall image quality ratings between the VFA and CFA scans. An optimized VFA scan can provide 65% of the SAR and 71% of the acquisition time of a CFA scan while being diagnostically equivalent. Lower SAR reduces heating, eliminates SAR pauses, and allows accelerated scans by reducing the TR. The time saved by faster HASTE acquisitions increases patient comfort and may be used to repeat scans with excessive fetal motion or to perform advanced sequences.

## 1 | Introduction

Fetal brain magnetic resonance imaging protocols typically include the acquisition of Half-Fourier Acquisition with Single-Shot Turbo Spin Echo (HASTE) stacks of slices in orthogonal sagittal, coronal, and axial views to obtain full-brain coverage [1–3]. If the fetus moves during the acquisition of a stack, the entire stack may need to be re-acquired to yield diagnostic images in all three views. Repeated HASTE scans can impose a heavy specific absorption rate (SAR) [2–9]. The minimum repetition time (TR) is also often restricted by the scanner SAR limits rather than by the time needed to acquire data. For example, a TR of 1400 ms may be used with ≈700 ms of data acquisition time followed by ≈700 ms of unused time to stay below SAR thresholds. A lower SAR scan not only reduces the heating that the subject perceives but also enables more efficient scanning by lowering the TR closer to the true data acquisition time.

One option to reduce the SAR is to reduce the flip angle of the refocusing pulses. For example, the default flip angle at our institution is 140°, but it may be lowered to 120° to reduce SAR without increasing TR (and thus the total scan time). However, lowering the flip angle uniformly across the refocusing pulses may lead to a reduced signal-to-noise ratio (SNR) and changes in contrast [10–12].

Another option to reduce SAR is to use a variable flip angle (VFA) pattern, in which the flip angle is varied over the refocusing pulses [10]. The flip angle pattern may be tailored to try to achieve a high signal at the center of the k-space with similar signal and contrast levels as a constant flip angle (CFA) scan but with reduced SAR. Compared to CFA patterns, tailored VFA patterns may also reduce T_2_ blurring due to slower T_2_ decay [12]. However, VFA scans can be more prone to signal dropout due to motion [13].

VFA strategies have been used to reduce SAR in T2-weighted Fast (Turbo) Spin Echo (FSE/TSE) and HASTE applications such as abdominal [14–16] and brain [11, 17–21] imaging. Different methods have been used to select the flip angles. For example, Loening et al. performed a sweep of the flip angle parameter space in simulation to select values that balanced minimizing TR and SAR while maintaining SNR and contrast [14]. Similarly, Wu et al. simulated a range of flip angle combinations to select the set with the highest contrast within signal intensity and SAR constraints [22]. Herrmann et al. performed an initial volunteer study of different flip angle patterns and used image quality ratings from radiologists to identify the pattern with the lowest SAR that still provided acceptable image quality [15]. Another type of approach that has been proposed in the literature is an analytical optimization of the flip angle pattern. For example, Zhao et al. used a global optimization algorithm to optimize the full flip angle pattern and filtering parameters to control T2 blur [17]. In some patterns, an additional constraint was placed on the optimization to limit SAR. Keerthivasan et al. used a genetic algorithm to solve a multi-constraint optimization that aimed to improve T2 estimation in the abdomen while constraining for SNR, contrast, and SAR [16].

Recently, Arefeen et al. explored VFA patterns to reduce SAR in fetal brain HASTE imaging [19–21]. However, they noted reduced gray-white matter contrast-to-noise ratio (CNR) in the VFA images compared to the CFA images. Recent work [21] aimed to maximize the gray and white matter contrast at the echo time (TE). The problem was posed as an optimization without additional constraints, and the optimization was solved using a gradient descent algorithm. The optimization resulted in improved CNR compared to a previous heuristic VFA pattern [20], although CNR was still slightly lower than the CFA scan. The VFA pattern enabled reduced SAR and TR compared to the CFA scan. Inspired by the work pioneered by Arefeen et al., we aimed to design an optimized VFA pattern for fetal brain imaging that minimizes the signal differences between VFA and CFA acquisitions.

In this work, a variable flip angle pattern for fetal HASTE imaging is optimized to minimize the expected signal difference over the full echo train between the proposed VFA scan and the standard CFA scan, while constraining the SAR and using a reduced TR. Our hypothesis was that by minimizing the expected signal differences, similar image quality to the CFA scan could be achieved while constraining only for the target metric (SAR). Extended phase graph simulations with estimated relaxation times for fetal brain tissues were used to predict signal evolutions in the optimization. The proposed VFA pattern was evaluated in simulations, in a phantom, and in healthy pregnant volunteers. The proposed pattern can be used to speed up fetal HASTE acquisitions while still providing reduced SAR compared to the CFA option. Reduced SAR also improves patient comfort by reducing heating, which is frequently reported by patients who undergo repeated HASTE scans.

## 2 | Methods

### 2.1 | Flip Angle Optimization

The variable flip angle scan was designed to achieve a SAR of less than or equal to 65% of the constant flip angle scan. Preliminary testing (not shown) suggested that this SAR reduction may be feasible without compromising image quality. SAR for a single TR was calculated as:

(1) $$\mathrm{SAR}\propto \frac{\sum_{n=1}^{\mathrm{number of flip angles}}\alpha_{n}^{2}}{\mathrm{TR}}$$

where α is the flip angle and the number of flip angles includes the initial 90° excitation pulse and the refocusing pulses. Note that this is a simplified equation for SAR and does not include all of the factors that contribute to SAR, such as the Larmor frequency and patient characteristics. These additional factors are expected to be constant between the CFA and proposed VFA scans.

The VFA scan was designed with a TR of 1000 ms rather than 1400 ms, which is typically used at our institution, such that the acquisition time of the VFA scan is 71% of the acquisition time of the CFA scan. Note that according to Equation (1), using a shorter TR will increase the SAR if the flip angles are not reduced correspondingly. For example, to achieve a scan with 65% of the SAR and 71% of the acquisition time using constant flip angles, the refocusing pulses would need to be reduced from 140° to about 95°. A variable flip angle pattern can be described by four control points—**α** = [α_start_, α_minimum_, α_center_, α_end_]—as detailed by others [13–16]. The control points describe the flip angle pattern used to acquire all echoes in each TR. For a given pattern, the expected signal over the echoes was simulated using an extended phase graph (EPG) formalism [10]. The relaxation values used for fetal gray and white matter in all simulations were: T_1, gray matter_ = 2500 ms; T_2, gray matter_ = 162 ms; T_1, white matter_ = 3300 ms; T_2, white matter_ = 232 ms [23].

The VFA control points were selected by minimizing the difference between the expected signal from a CFA scan at 140° with TR = 1400 ms and from the VFA accelerated, reduced-SAR scan:

(2) $$\min_{\alpha }{\left\|S_{\mathrm{VFA}}\left(\alpha \right)-S_{\mathrm{CFA}}\left(140^{{}^{\circ}}\right)\right\|}_{2}\;\mathrm{s.t.}\;{\mathrm{SAR}}_{\mathrm{VFA}}\leq 0.6475x{\mathrm{SAR}}_{\mathrm{CFA}}$$

where **α** is the set of the four control points, *S_VFA_* is the simulated signal evolution using the variable flip angle pattern, *S_CFA_* is the simulated signal evolution from using a constant flip angle of 140°, *SAR_VFA_* is the SAR of the variable flip angle scan, and *SA R_CFA_* is the SAR of the constant flip angle scan. The SAR of the VFA flip angle pattern was constrained to 64.75% of the CFA pattern (the slight reduction from 65% was to accommodate rounding the final flip angle pattern to integers, as expected in the sequence user interface). The signal evolutions from gray and white matter were concatenated together for each condition. This formulation was intended to minimize the difference in signal evolution across all refocusing echoes for both tissue types.

The optimization was solved using the *fmincon* function in MATLAB (version 2022a; MathWorks, Natick, MA). *fmincon* was run with the “central” finite difference type, a maximum of 10,000 function evaluations, a maximum of 2000 iterations, and a constraint tolerance of 1 × 10^−5^. Lower and upper bounds of the four control points were set to [100°, 20°, 60°, 20°] and [180°, 180°, 180°, 180°], respectively.

Following a basin-hopping strategy [24], the minimization was run 10,000 times with different starting points selected using MATLAB’s *rand* function. Only starting points that initially satisfied the bounds and SAR constraint were used. The resulting output control points, exit flag, and objective function were saved for each run. Only successful runs (exit flag > 0) were considered. Of these, the final control point set was selected from the top 10 (lowest objective function) runs. Note that in practice, the top 10 runs differed negligibly, and after rounding the flip angles to integers, all 10 control point sets were the same.

VFA data were acquired using a prototype sequence package for HASTE imaging from the scanner vendor that can prescribe a variable flip angle pattern from four control points [13, 15].

The current fetal brain HASTE protocol at our institution uses the following parameters: TR/TE: 1400 ms/99–100 ms; FOV: 256 × 256 mm^2^; in-plane resolution: 1 × 1 mm^2^; phase partial Fourier factor: 4/8; phase oversampling: 0%–30%; slice thickness: 2–3 mm; slice gap: 0%; interleaved slice acquisition (interleave step: 2 [CFA] or 5 [VFA]); flip angle: 140°. Data are acquired with a Cartesian acquisition, and an in-plane reduction factor of 2 is used (uniform undersampling in the phase encode direction). Fully sampled calibration data for a parallel imaging (GRAPPA [25]) reconstruction are acquired in the center of k-space as part of the single-shot acquisition. The number of slices per stack is adjusted for full fetal brain coverage. Aside from the TR and flip angle modifications described above and the noted difference in interleave step, all other parameters of the VFA scan matched the CFA scan. The same image reconstruction pipeline was used for both.

The expected signal evolutions from the proposed VFA and CFA at 140° scans with these parameters were simulated using EPG calculations.

### 2.2 | Simulations

The EPG-simulated VFA and CFA signal evolutions were compared using different metrics: SAR, signal, area under the signal decay curve (AUDC), relative contrast, resolution, and sensitivity to motion. SAR was calculated as described in Equation (1). Signal was calculated as the magnitude of the signal at the center echo of k-space. AUDC was calculated as the signal magnitude at each echo time multiplied by the echo spacing, summed over all echoes. Relative contrast was calculated as the difference between white matter (WM) and gray matter (GM) signal magnitude over white matter signal magnitude at the center echo of k-space.

Resolution was evaluated by calculating the full-width-at-half-maximum (FWHM) of the point spread function. The corresponding modulation transfer function (MTF) was taken to be the sampling pattern in the phase-encode direction modulated by the simulated signal. Partial Fourier sampling in the MTF was corrected using conjugate symmetry, and undersampling was handled by filling unsampled lines with the following sampled line. Note that this FWHM calculation does not fully replicate the image reconstruction pipeline, which uses parallel imaging to resolve the undersampling.

The effect of constant-velocity bulk motion in one direction was simulated by incorporating eqs. 53 and 54 in Weigel’s review of extended phase graphs [26] into the EPG simulation. Motion was simulated as entirely in one direction, parallel to the direction of spatial encoding, since only the motion components that are parallel or anti-parallel will have an effect in the 1D EPG simulation [26]. Motion up to 4 mm/s was simulated, following the “strong” motion amplitude simulated by Lajous et al. in their FaBiAN phantom [23], where strong motion was considered up to 4 mm of translation along each axis per slice. The signal magnitude at the center of the k-space with simulated motion was recorded.

SAR, signal, AUDC, relative contrast, resolution, and motion sensitivity were compared between the CFA and proposed VFA scans.

A grid search over VFA control points was also performed to better understand the optimization search space. To reduce the total search space, the start and end control points were fixed to the results of the minimization (α_start_ = 159°, α_end_ = 42°). The middle two points, α_minimum_ and α_center_, were incremented in 1° steps between the lower and upper bounds set in the minimization. The EPG-simulated signal evolution at each control point set was saved, and the same image metrics described above were calculated.

All simulations were performed in MATLAB.

### 2.3 | Phantom Scans

An ISMRM/NIST mini system phantom (model 136, CaliberMRI, Boulder, CO) [27] was scanned using the CFA and proposed VFA sequence. A stack of 20 slices centered over the T_2_ layer of the phantom was acquired 100 consecutive times for multi-replica SNR and CNR measurements [28, 29]. All measurements were performed on the scanner-output DICOM images. SNR maps were calculated as the pixel-wise mean of the signal over the 100 frames divided by the pixel-wise standard deviation of the signal.

The phantom T_2_ layer contains multiple vials, but none completely match the T_1_/T_2_ values of fetal gray/white matter. Therefore, the vials that were the closest were selected for further analysis. Vial T2–3 has T_1_/T_2_ values of 2119.34/287.48 ms, respectively, at 24°C and was selected to represent fetal white matter (taken to be T_1_/T_2_ = 3300/232 ms at 3 T^23^). Vial T2–4 has T_1_/T_2_ values of 1715.06/187.21 ms and was selected to represent fetal gray matter (taken to be T_1_/T_2_ = 2500/162 ms at 3 T [23]).

A single slice centered over the T_2_ layer from the stack was selected, and regions of interest (ROIs) of 175 pixels were drawn on the SNR maps. SNR is reported as the mean values within the ROIs. CNR is calculated as the difference in SNR between the two vials.

The last frames from each 100-measurement scan were also analyzed for signal and relative contrast. The same ROIs were drawn on these images. Signal is given as the mean value within the ROIs, and relative contrast was calculated as described above in the simulation.

The last frames were additionally used to compare the spatial resolution of the CFA and VFA scans. Line profiles were drawn through the two vials of interest in both the readout and phase-encode directions and visually compared.

### 2.4 | In Vivo Scans

Data were acquired on a 3-T scanner (MAGNETOM Prisma, Siemens Healthineers, Forchheim, Germany) from 19 healthy pregnant volunteers who were prospectively recruited as part of a HIPAA-compliant study. The study was approved by the Institutional Review Board (IRB), and written informed consent was obtained from all participants. The CFA at 140° and the proposed VFA scans were run in each of three views (sagittal, coronal, and axial, with respect to the fetal brain) using the above-mentioned parameters. Slice positions were matched between CFA and VFA scans in every view. In cases where one scan had significant motion (e.g., CFA) and the other did not (e.g., VFA), the problematic sequence (e.g., CFA) was reacquired to ensure that any differences were due to the technique itself rather than motion.

For the first 10 cases, the scanner-reported 6-min averaged whole-body SAR (hereafter referred to as “relative SAR” because the reported SAR is given as relative to the SAR limit of 2 W/kg), measurement time, and pause after measurement due to SAR limits were recorded. The total scan time was calculated as the sum of the measurement and pause times. Relative SAR, measurement time, and total scan time were compared between the CFA and VFA scans using paired t-tests (normally distributed variables) or Wilcoxon ranked sum tests (not normally distributed variables). Normality was assessed using Shapiro–Wilk tests. All statistics were calculated in R.

### 2.5 | Quantitative Image Evaluation

For every stack of CFA and VFA acquisitions, manual ROIs were drawn on the periventricular white matter and cortical gray matter. These segmentations were used to calculate gray matter and white matter SNR and gray/white CNR, with the gray or white matter volumes used as signal ROIs and a noise ROI drawn in a region of air outside of the maternal body. SNR was calculated as the mean value of signal in the signal ROI divided by the standard deviation of noise in the background (air) ROI. CNR was calculated as the difference between the mean signal values in the white and gray matter ROIs divided by the standard deviation of noise in the background (air) ROI. Noise values were divided by a 0.66 correction factor before calculating SNR and CNR to account for the Rayleigh distribution of noise in air regions of magnitude images [30]. Mean values of SNR and CNR were calculated, and differences in SNR and CNR between CFA and VFA scans were compared using a linear mixed effects model.

### 2.6 | Qualitative Image Evaluation

Three radiologists, C.J., S.F., and C.A., evaluated the image quality of the 2D image stacks. Each image set for the 2D assessment included sagittal, coronal, and axial views for a subject with either the CFA or the proposed VFA scan. Each radiologist evaluated the images using a 5-point Likert scale (Table S1), providing an independent rating for each view.

To minimize bias in image assessment and maintain the study’s blindness, we utilized a block randomization strategy. Image sets were organized into three distinct groups—each containing an equal number of images from both MRI sequences—and were assessed sequentially. Importantly, image sets in a group were not necessarily from the same subject, and MRI sequences were presented in a random order, which helped prevent any sequence-specific or subject-specific bias.

Fetal motion that can vary between scans may affect a direct comparison of the individual slices within 2D HASTE stacks. Therefore, in addition to the qualitative evaluation of the original HASTE stacks, super-resolution slice-to-volume reconstruction [31–33] was used to reconstruct a motion-compensated volumetric (3D) image of every fetal brain from its set of three orthogonal stacks. A super-resolution 3D image was reconstructed for each of the CFA and VFA sequences. The radiologists evaluated these 3D reconstructions using the same methodology as above.

We conducted a comparative analysis of quantitative and qualitative evaluations of images from the CFA and VFA scans using mixed-effects models. To account for intra-rater and inter-subject variability, the quantitative image evaluation model considered random effects for “subject,” and the qualitative image evaluation model considered random effects for “subject” and “rater.” After fitting the models, we used Tukey’s honest significant difference (HSD) test to perform post hoc pairwise comparisons and determine statistically significant differences between the methods. Inter-rater reliability was evaluated using Fleiss’ kappa statistic, which quantifies the agreement between raters beyond chance.

## 3 | Results

### 3.1 | Flip Angle Optimization

The *fmincon* optimization to minimize signal differences between the proposed VFA flip angle pattern and the CFA flip angle pattern was run with 10,000 different starting points. Ninety-eight percent of the runs were successful (exit flag > 0), and the top 10 results yielded control points of **α** = [α_start_ = 159°, α_minimum_ = 133°, α_center_ = 143°, α_end_ = 42°].

**FIGURE 1 |:**
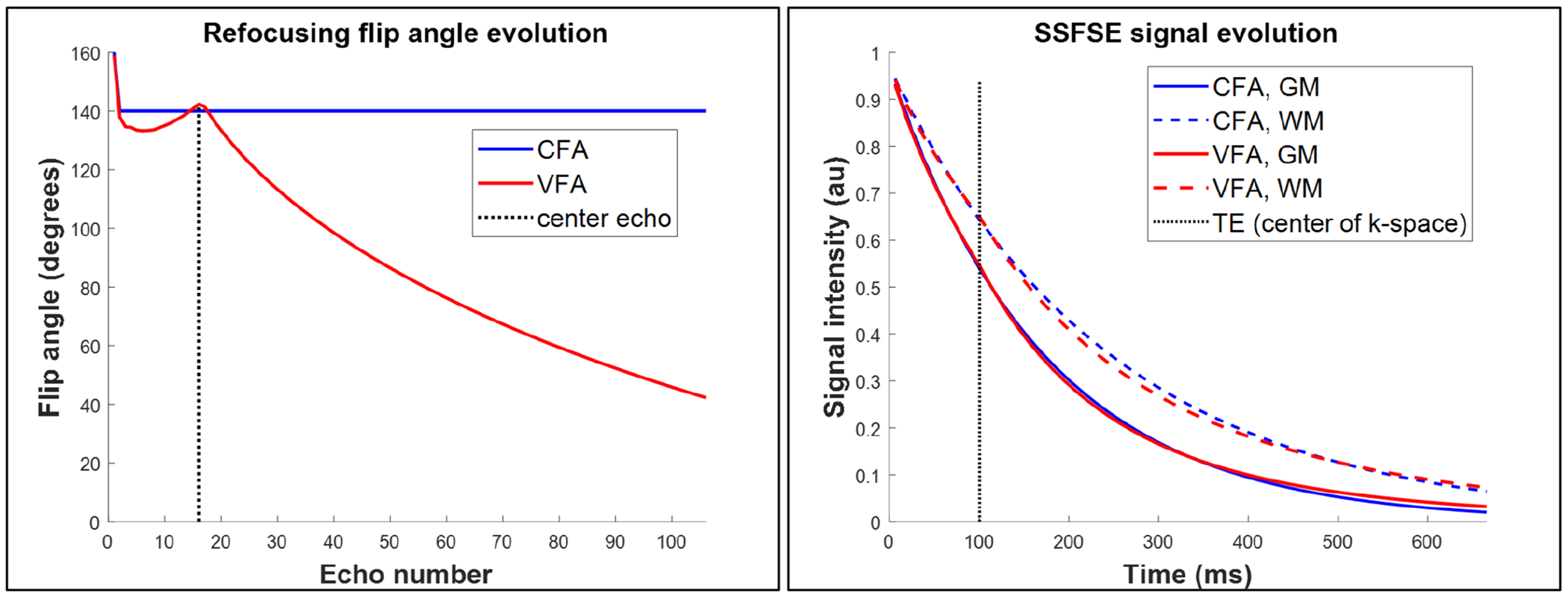
Proposed variable flip angle pattern and simulated signal evolutions. Refocusing flip angles (left) and expected signal magnitude (right) over the echo train for the proposed VFA and CFA scans. The dashed vertical line denotes the echo at the center of k-space, which is the same for both scans. Note that the HASTE acquisition is a single shot acquisition, and the entire echo train is acquired in one TR. Signal evolution was estimated using the extended phase graph formalism. CFA: constant flip angle; GM: gray matter; VFA: variable flip angle; WM: white matter.

Figure 1 shows the flip angle patterns over all echoes for the proposed VFA and CFA sequences. The expected signal evolutions from EPG simulation are also shown. The signal evolutions show similar signal levels at the center of k-space, with differences in later echoes.

### 3.2 | Simulations

Table 1 shows the expected performance of the CFA and proposed VFA scans in terms of different image metrics. The VFA scan has 63.7% of the SAR of the CFA scan, meeting the 65% constraint. The VFA scan has 98.9% of the gray/white matter contrast of the CFA scan (0.16 vs. 0.162 in arbitrary units, respectively). The VFA scan has approximately the same AUDC for gray matter as the CFA scan (0.161 au vs. 0.16 au, respectively) and 98% of the AUDC for white matter (0.215 au vs. 0.219 au). The VFA scan is expected to have a slightly higher signal at the center of k-space for both gray and white matter (gray matter: 0.545 au vs. 0.539 au; white matter: 0.649 au vs. 0.643 au). The two scans have the same FWHM for white matter, and the VFA scan has a better FWHM compared to the CFA scan for gray matter (2.75 vs. 3.125 pixels). The VFA is slightly more sensitive to motion compared to the CFA scan. Motion sensitivity is given as the percentage of the no-motion signal preserved at the center of the k-space when motion of 4 mm/s velocity along one axis is simulated. The CFA scan preserves 99.7% of the signal for both gray and white matter, while the VFA scan preserves 99.3% of the signal for both gray and white matter.

Results of the grid search are shown as heat maps in Figures S1–S5. Based on those results, SAR increases with both increasing α_min_ and α_cent_. Relative contrast appears more dependent on the value of α_min_ than on α_cent_, where a higher α_min_ yields higher relative contrast. Signal appears dependent on both α_min_ and α_cent_, where decreasing α_min_ and increasing α_cent_ yield a higher signal. The AUDC shows a nonlinear relationship with α_min_ and α_cent_. Lower α_min_ values appear to yield higher AUDC, although the effects are not equal between gray and white matter. FWHM depends on both α_min_ and α_cent_, where increasing either one increases the FWHM. Finally, sensitivity to motion appears most dependent on α_min_, where increasing α_min_ improves motion robustness.

These maps help to visualize the trade-offs between different image metrics. For example, increasing α_min_ appears to improve motion sensitivity but also increases SAR, while decreasing α_min_ to improve AUDC may also reduce the relative contrast.

### 3.3 | Phantom Scans

**FIGURE 2 |:**
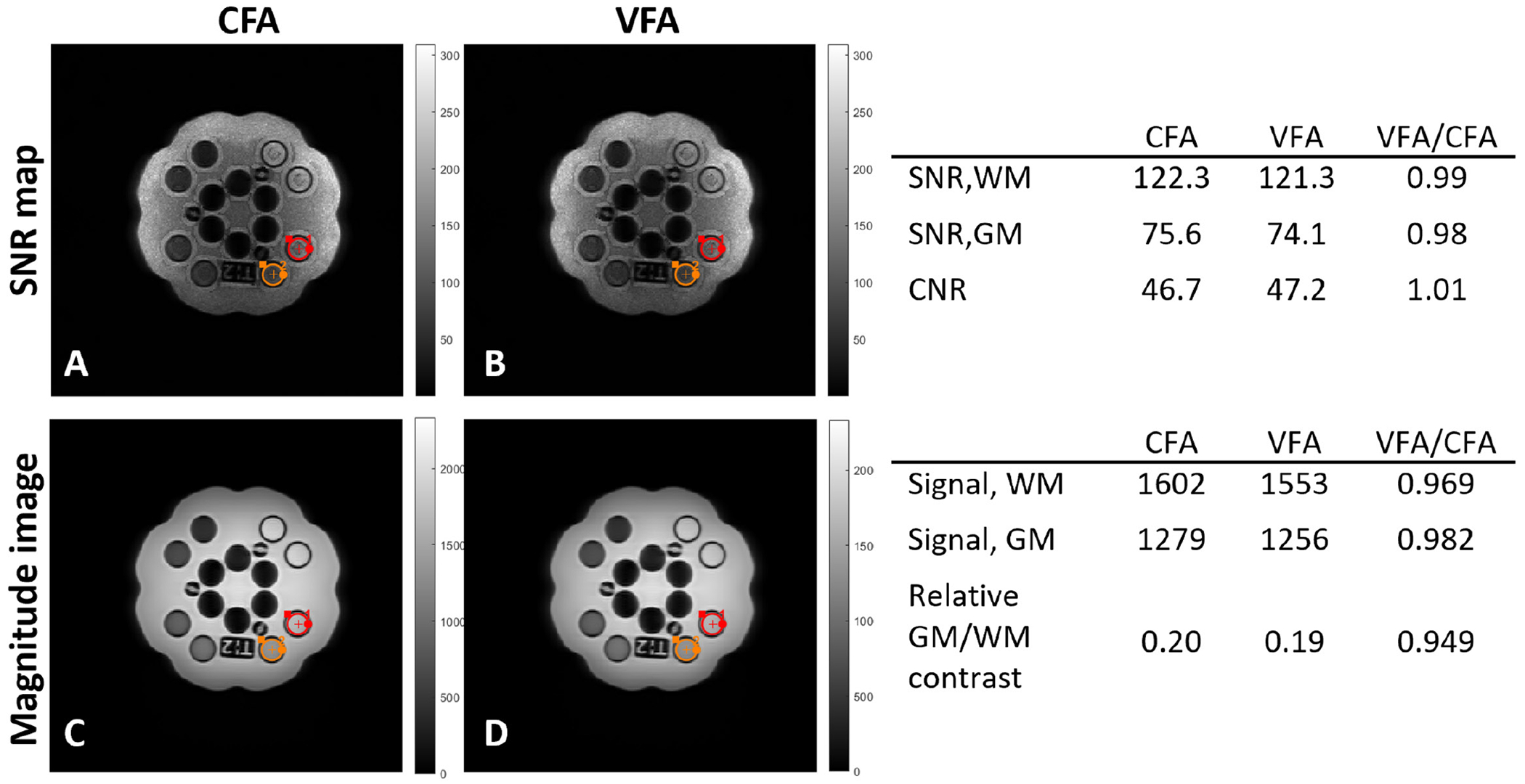
Signal and contrast measurements in the ISMRM/NIST system phantom. SNR maps (A, B) and magnitude images (C, D) of the T_2_ layer in the ISMRM/NIST standard phantom imaged with the CFA scan (A, C) and the proposed VFA scan (B, D). ROIs are drawn in the vials with T_1_/T_2_ values closest to fetal gray matter (orange ROIs) and white matter (red ROIs). The mean values for the SNR, CNR, signal, and relative contrast of the two scans are shown on the right. The VFA/CFA ratios for SNR and CNR indicate only small differences between the scans. CFA: constant flip angle; CNR: contrast-to-noise ratio; GM: gray matter; SNR: signal-to-noise ratio; VFA: variable flip angle; WM: white matter.

Figure 2 shows SNR maps and magnitude images from the T_2_ layer of the ISMRM/NIST system phantom, imaged with the CFA and proposed VFA scans. The CFA scan yielded higher SNR for both white and gray matter compared to the VFA scan (white matter: 122.29 vs. 121.33; gray matter: 75.616 vs. 74.118 for CFA and VFA scans, respectively), but the VFA scan yielded higher CNR (46.676 vs. 47.207 for CFA and VFA, respectively).

In the magnitude images, the CFA scan showed a higher signal in both gray and white matter and higher relative contrast (white matter: 1602.3 vs. 1553.2; gray matter: 1279.3 vs. 1256; relative contrast: 0.202 vs. 0.191). The largest difference between the CFA and VFA scans was in the relative contrast, where the contrast of the VFA scan was 94.9% of that of the CFA scan. Although the T_1_/T_2_ values of the phantom vials did not perfectly match the expected in vivo fetal gray matter/white matter relaxation parameters, a simulation of image metrics using the phantom parameters showed that the expected relative CFA/VFA performance was similar to what was simulated for fetal parameters (Table S2).

Figures S6 and S7 show the line profiles drawn through the vials of interest. In both the readout and phase encode directions, the widths of the signal peaks and drops visually appear to match. In the phase encode direction, the CFA scan shows slight overshoots and undershoots at bright/dark signal edges.

### 3.4 | In Vivo Scans

**FIGURE 3 |:**
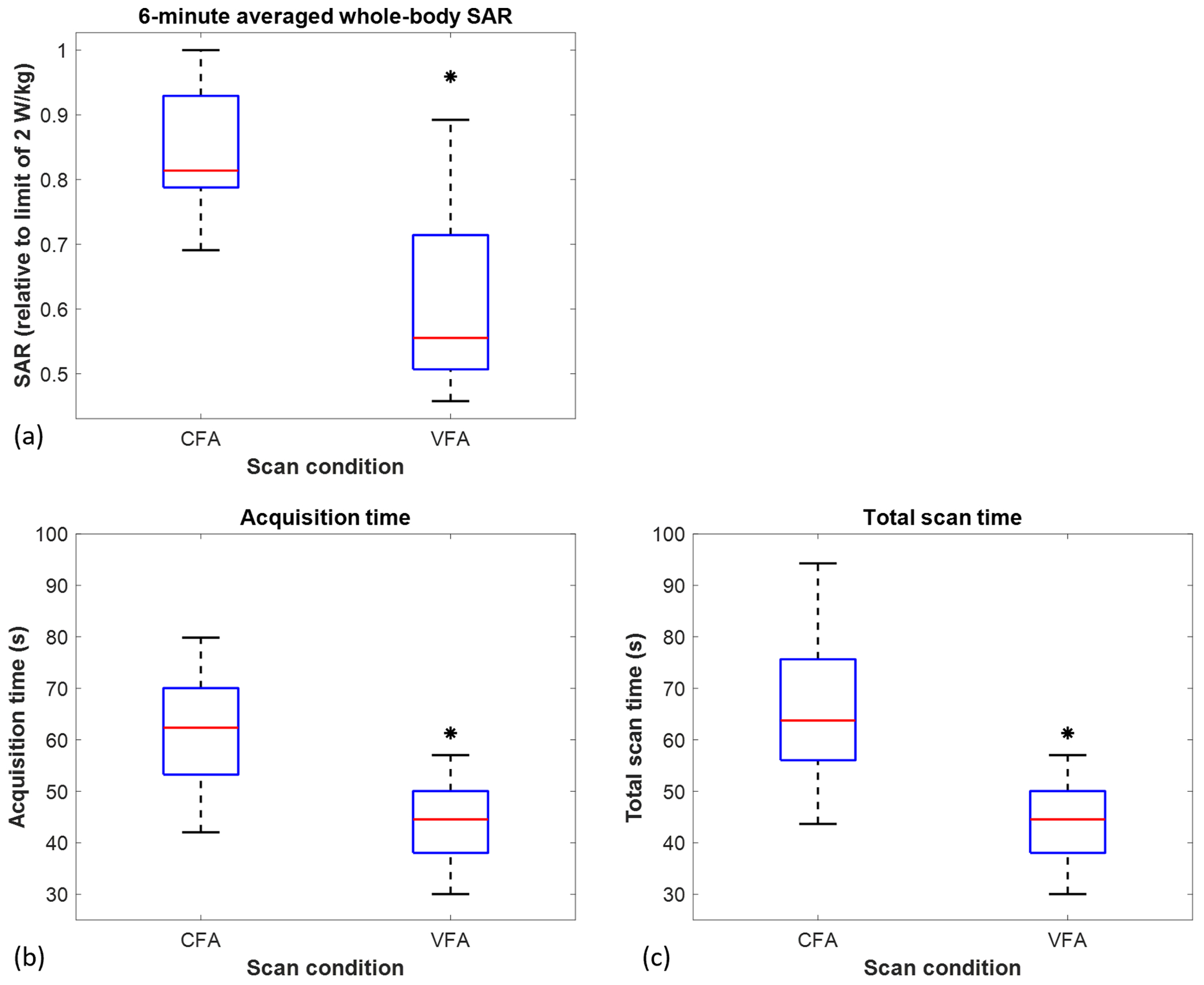
SAR and acquisition time for in vivo fetal imaging. (a) SAR is given as relative to the scanner limit of 2 W/kg. (b) Acquisition time is in seconds and has been reported for a VFA scan with TR = 1 s, compared to the baseline CFA with TR = 1.4 s. (c) Total scan time is in seconds and is the sum of the measurement time and any pause time inserted by the scanner to avoid SAR limits. Black asterisks denote significant differences between the CFA and VFA scans. The VFA scan can provide 65% of the SAR of the CFA scan with a faster acquisition. CFA: constant flip angle; SAR: specific absorption rate; VFA: variable flip angle.

The statistics of the relative SAR values and the scan measurement times from the first 10 cases are shown as boxplots in Figure 3. The median relative SAR when scanning with the CFA scan was 0.814, while the median relative SAR when scanning with the VFA scan was 0.555. The CFA scan reached the SAR limit in some cases, and a pause time was automatically introduced by the scanner after the measurement. In these cases, the relative SAR for the CFA scan was near or at 1, and the relative SAR for the VFA scan was higher than the expected 0.65, but the VFA SAR savings appeared to avoid the need for a pause. The median acquisition times for the CFA and VFA scans were 62.3 and 44.5 s, respectively, and the median total scan times (acquisition time + SAR pause times) were 63.7 and 44.5 s, respectively. The scanner enforced pauses in 14 of the 30 CFA scans and none of the VFA scans. The SAR delays with the CFA scans increased the upper quartile of the scan time by ≈5 s, and the median scan time by ≈1.4 s.

Values were statistically significantly different between the CFA and VFA scans for all three metrics (*p* < 0.05). Relative SAR was compared using a Wilcoxon ranked sum test as the values were not normally distributed, and the *p*-value was 1.55 × 10^−8^. Measurement and total scan times were compared using paired *t*-tests, and the resulting *p*-values were 1.26 × 10^−23^ and 2.63 × 10^−15^, respectively.

### 3.5 | Quantitative Image Evaluation

**FIGURE 4 |:**
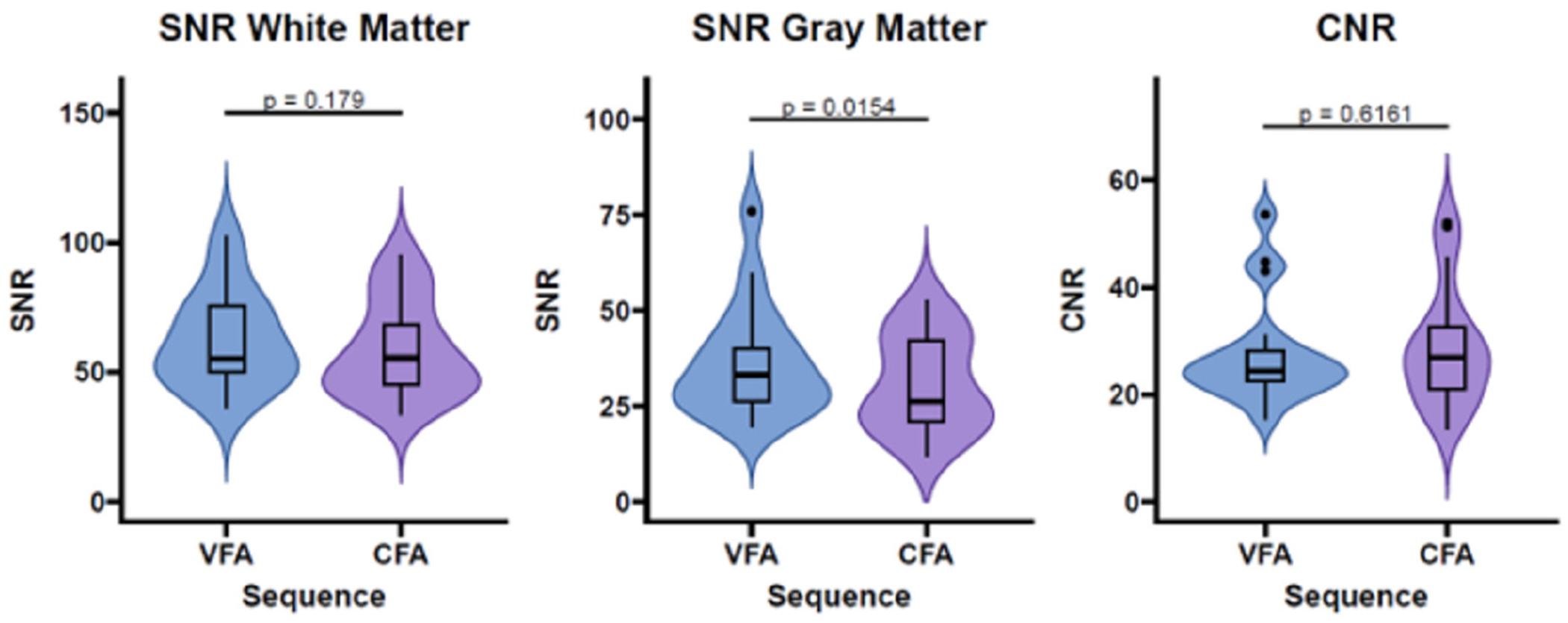
Image metrics calculated for the constant flip angle (CFA) and proposed variable flip angle (VFA) scans on the in vivo volunteers. *p* values indicate whether differences between the scans are statistically significant. The variable flip angle VFA scan showed a higher signal-to-noise ratio (SNR) for both gray and white matter compared to the CFA scan; however, only the SNR of gray matter was significantly different. The contrast-to-noise ratio (CNR) remained comparable between the two methods.

The results show that the VFA scan provided higher SNR for both gray and white matter compared to the CFA scan; however, there were only significant differences in the SNR of gray matter (*p* = 0.0154), with the VFA scan having an average higher SNR. The CNR remained comparable between the two methods. Figure 4 summarizes the SNR and CNR values of the 19 volunteers between the CFA and VFA scans. Mean CNR values and gray matter and white matter SNR values for the VFA and CFA scans can be found in Table 2.

### 3.6 | Qualitative Image Evaluation

Inter-rater reliability for the individual stacks was 0.346 (fair agreement), and for the 3D reconstructions, it was 0.662 (substantial agreement). Intra-rater reliability ranged from 0.600–0.913 (substantial to almost perfect agreement). Based on the reliability results, scores from the three radiologists were combined by calculating the median score for each stack to avoid including outlier ratings.

**FIGURE 5 |:**
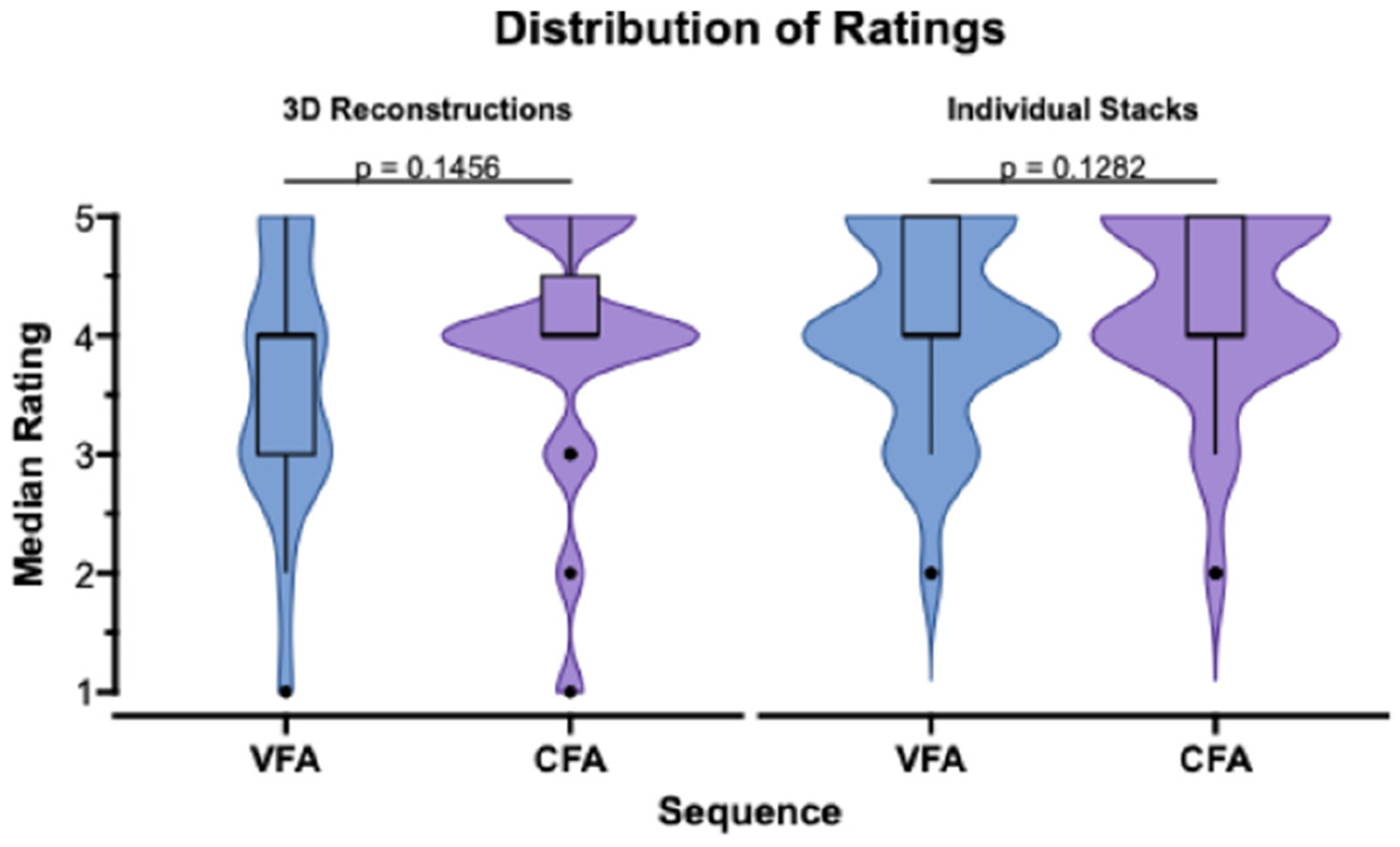
Distributions of the radiologists’ ratings of the in vivo images. Ratings are grouped for the individual stacks and the 3D reconstructions of the CFA and proposed VFA scans. *p* values are shown to assess if there are significant differences between the scans. CFA: constant flip angle; VFA: variable flip angle.

**FIGURE 6 |:**
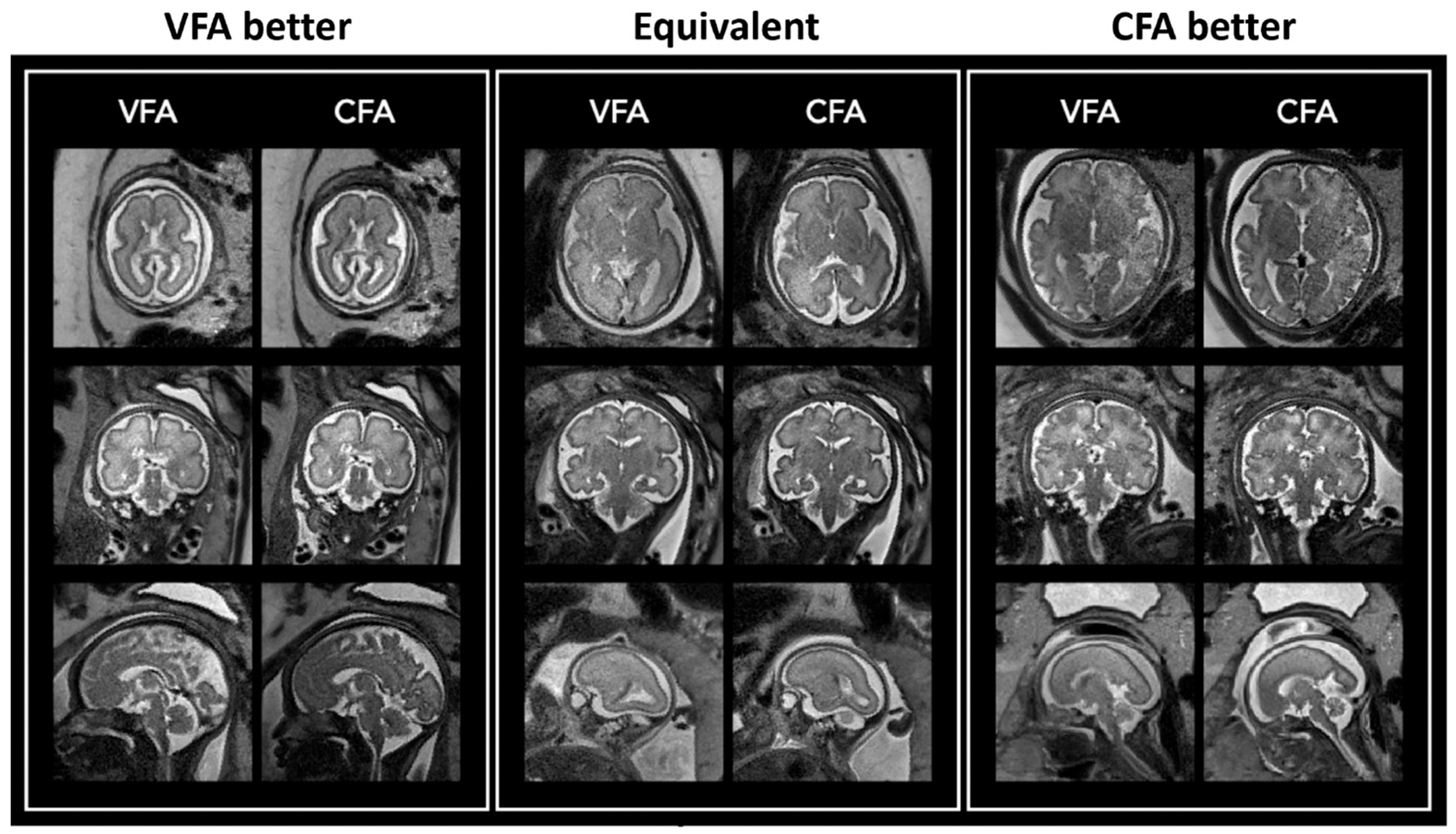
Example 2D HASTE in vivo images. Example coronal, axial, and sagittal 2D images from volunteers in which the ratings of the radiologists were better for the VFA scans (left column), were equivalent (middle column), and were better for the CFA scans (right column). CFA: constant flip angle; VFA: variable flip angle.

**FIGURE 7 |:**
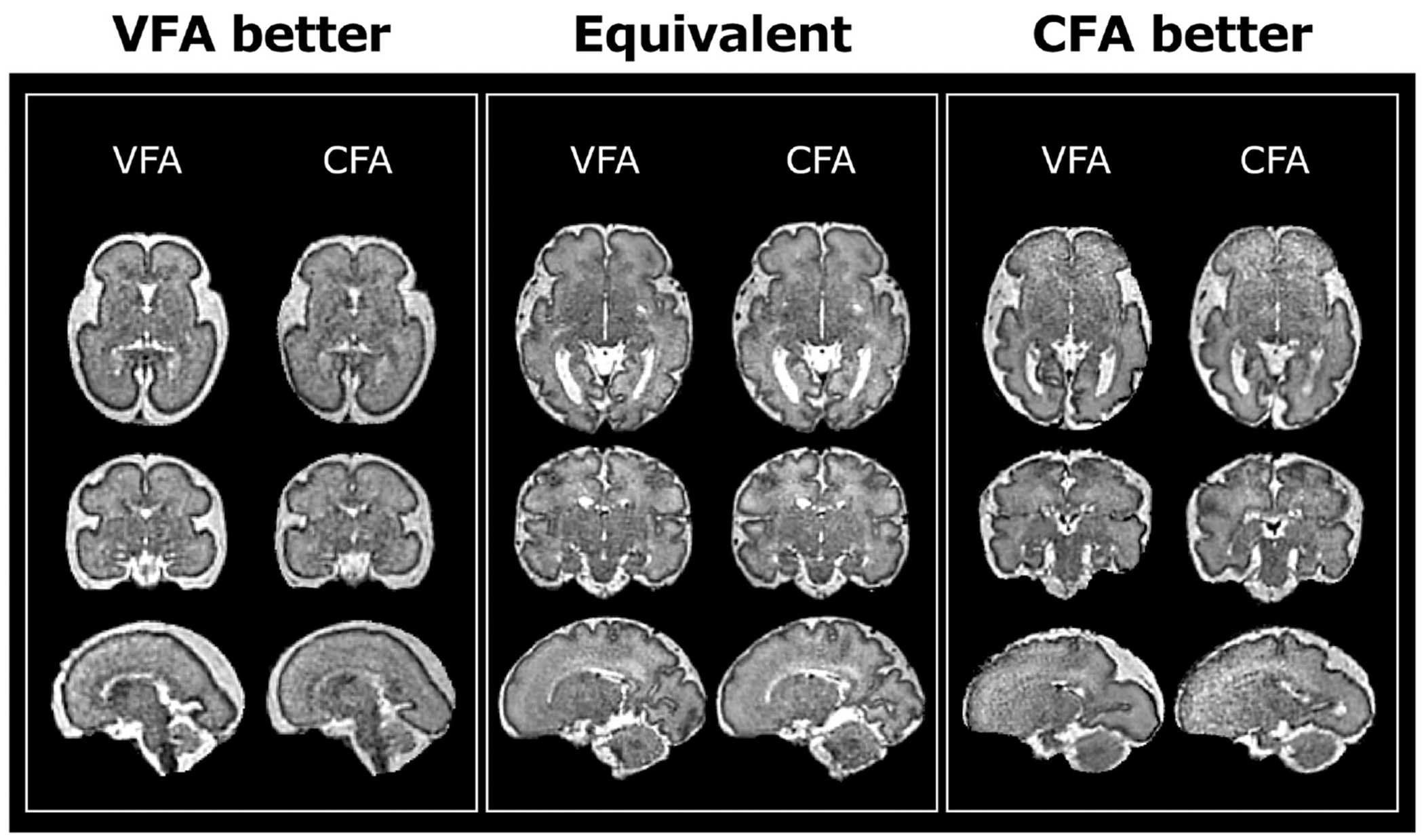
Example super-resolution slice-to-volume reconstructions of 2D HASTE in vivo images. Example 3D reconstructions from volunteers in which the radiologist ratings of the reconstructions were better for the VFA scans (left column), were equivalent (middle column), and were better for the CFA scans (right column). CFA: constant flip angle; VFA: variable flip angle.

The mean ratings for the 2D CFA and VFA stacks were 4.26 and 4.07, respectively, with no significant differences between the techniques. The mean ratings for the 3D CFA and 3D VFA were 3.89 and 3.58, respectively, with no significant differences between the techniques. Figure 5 shows the distributions of the radiologists’ ratings with violin plots and the respective *p*-values. Figure 6 shows example images from the 2D CFA and VFA HASTE stacks that the radiologists scored. Axial, coronal, and sagittal images are shown from different volunteers in three different columns where radiologists preferred the VFA, agreed that VFA and CFA were equivalent, and preferred the CFA. Figure 7 shows example images from super-resolution slice-to-volume reconstructions where the radiologists preferred the VFA reconstructions, agreed that the VFA and CFA reconstructions were equivalent, and preferred the CFA reconstructions.

## 4 | Discussion

An optimized variable flip angle pattern is proposed in this article for fetal brain HASTE imaging. Compared to the standard constant flip angle scan, the VFA scan offers a lower SAR, which can be used to eliminate SAR delays and accelerate acquisitions by reducing the repetition time. The VFA scan has 65% of the SAR and 71% of the acquisition time of the CFA scan. Based on the in vivo assessment, the VFA scan is diagnostically comparable to the CFA scan. This work suggests that a VFA scheme can be used to speed up fetal HASTE imaging while reducing the SAR without significant compromises in image quality, as demonstrated by quantitative and qualitative evaluation in healthy pregnant volunteers.

In this work, the SAR constraint and TR target for the optimized VFA scan were chosen to enable modest savings over the CFA scan while maintaining image quality. We did not attempt to minimize the TR to the SAR-limited value for each volunteer and instead scanned with a lower SAR for reduced heating. However, this optimization framework could be combined with other SAR or time-reducing techniques, such as higher reduction (acceleration) factors combined with advanced reconstructions like deep learning, to target greater savings.

Compared to similar previous works investigating VFA for fetal brain HASTE imaging [19–21], here we pose a different optimization strategy to minimize the expected signal difference between the CFA and VFA scans over the full signal evolution rather than maximizing the VFA contrast at the echo time. It was hypothesized that this strategy would yield more similar image quality between the CFA and VFA scans. Additionally, an approximation of motion-induced signal drops was included in simulations. From simulations, it was expected that the proposed VFA scan would have a higher signal and comparable contrast to the CFA scan and similar motion sensitivity.

Note that in this work, the signal magnitude at the center echo of k-space in EPG simulation was used to predict signal and relative contrast. Other works have instead simulated circular or rectangular objects and measured signal within ROIs [14, 16]. This approach may better predict on-scanner performance and should be explored in future iterations of this optimization.

This work did not explore the effects of the proposed VFA pattern on magnetization transfer (MT). Weigel et al. previously described fewer magnetization transfer-induced signal attenuations when using low constant or variable flip angles as compared to high constant flip angles in turbo spin echo sequences [34]. Future work should examine the impact of MT on multi-slice HASTE imaging of the fetal brain and any changes in signal and contrast due to the proposed VFA pattern compared to a CFA acquisition.

The ISMRM/NIST system phantom [27] provided a controlled way to compare the CFA and VFA scans without fetal motion, as random fetal motion hampers direct comparison between scans. However, the relaxation properties of the phantom differed from those used in optimizing the scan and from those that are expected in vivo. The differences may partly account for the differences in simulated versus phantom signal and relative contrast values. Future work may use a dedicated fetal phantom with gray/white matter components, although such phantoms are not currently widely available to the best of our knowledge. In addition, the tissue properties and the relaxation times of fetal GM and developing WM are expected to change throughout gestation, which may require fine-tuning the VFA schedule at different gestational ages for optimized performance. Ultimately, the VFA flip angle schedule is one component of the fetal neural imaging protocol. Other factors, such as the degree and frequency of fetal motion and image SNR, which vary depending on the fetal pose, fetal and maternal position, coil coverage, and RF penetration and standing wave artifacts [2], may dictate further protocol changes for improved imaging.

In this study, motion sensitivity was investigated using simulations of translational motion along only one direction and was used as a comparison tool between different scans. In practice, the fetal head may exhibit rigid body motion with translation and rotation along all three axes at varying velocities. More rigorous phantom experiments with more realistic motion trajectories could be explored in future studies to better characterize the sensitivity of the VFA scan to fetal motion. A VFA scan with acceptable motion tolerance can also be combined with pipelines that automatically detect and re-acquire severely motion-corrupted images, such as that proposed by Gagoski et al. [35], providing robustness against mild and severe motion. A VFA scan that eliminates SAR delays and reduces the TR to accelerate slice acquisitions may also boost the performance of methods that aim to automatically estimate the fetal pose, track fetal motion, and prospectively navigate slice positions during acquisition [36–38].

## 5 | Conclusions

This work proposes an optimized variable flip angle (VFA) pattern for fetal brain HASTE imaging with reduced SAR and acquisition time. The control points of the VFA pattern were selected to minimize the difference between the expected signal from a baseline constant flip angle scan and the expected signal from the VFA scan. The expected signal was estimated using the extended phase graph formalism considering fetal gray and white matter relaxation times. Simulations and phantom experiments showed minimal differences in contrast and signal. When tested in healthy volunteers, the proposed VFA scan was rated equal in terms of diagnostic quality to the standard constant flip angle scan, with 65% of the SAR and 71% of the acquisition time.

## Supplementary Material

Supplementary Material

Additional supporting information can be found online in the Supporting Information section.

## Figures and Tables

**TABLE 1 |:** Image metrics calculated from estimated signal evolutions for the CFA and proposed VFA scans.

Sequence	SAR (au)	Relative contrast (au)	Tissue	AUDC (au)	Signal (au)	FWHM (pixels)	Motion sensitivity (%)
CFA	1 (1.49 × 10^6^)	1 (0.162)	GM	1 (0.16)	1 (0.539)	1 (3.125)	1 (0.997)
			WM	1 (0.219)	1 (0.643)	1 (2.125)	1 (0.997)
VFA	0.637 (9.52 × 10^5^)	0.989 (0.16)	GM	1.008 (0.161)	1.012 (0.545)	0.88 (2.75)	0.996 (0.993)
			WM	0.98 (0.215)	1.01 (0.649)	1 (2.125)	0.996 (0.993)

*Note:* Signal for each scan was estimated using extended phase graph simulations. Metrics are normalized to the CFA values for better comparison between the two scans, and absolute values are shown in parentheses. The units of motion sensitivity are % of the signal without motion.

Abbreviations: AUDC: area under the decay curve; FMWH: full-width-at-half-maximum; GM: gray matter; SAR: specific absorption rate; WM: white matter.

**TABLE 2 |:** Quantitative evaluation of individual stacks.

Metric	VFA (Mean ± std)	CFA (Mean ± std)
SNR, WM	69.8 ± 35.9	63.0 ± 26.6
SNR, GM	42.2 ± 28.4	34.1 ± 18.5
CNR	27.6 ± 9.9	28.9 ± 11.3

Abbreviations: CNR: contrast-to-noise ratio; GM: gray matter; SNR: signal-to-noise ratio; WM: white matter.

## Data Availability

The data that support the findings of this study are available from the corresponding author upon reasonable request.

## Abbreviations:

AUDC: area under the signal decay curve
CFA: constant flip angle
CNR: contrast-to-noise ratio
EPG: extended phase graph
FWHM: full-width-at-half-maximum
GM: gray matter
HASTE: Half-Fourier Acquisition with Single-Shot Turbo Spin Echo
ROI: region of interest
SAR: specific absorption rate
SNR: signal-to-noise ratio
TE: echo time
TR: repetition time
VFA: variable flip angle
WM: white matter

## References

[1] Prayer D, Brugger PC, and Prayer L, “Fetal MRI: Techniques and Protocols,” Pediatric Radiology 34, no. 9 (2004): 685–693, 10.1007/s00247-004-1246-0. 15316689

[2] Gholipour A, Estroff JA, Barnewolt CE, , “Fetal MRI: A Technical Update With Educational Aspirations,” Concepts in Magnetic Resonance Part A 43, no. 6 (2014): 237–266, 10.1002/cmr.a.21321. PMC4515352 26225129

[3] Calixto C, Taymourtash A, Karimi D, , “Advances in Fetal Brain Imaging,” Magnetic Resonance Imaging Clinics of North America 32, no. 3 (2024): 459–478, 10.1016/j.mric.2024.03.004. 38944434 PMC11216711

[4] Hand JW, Li Y, Thomas EL, Rutherford MA, and Hajnal JV, “Prediction of Specific Absorption Rate in Mother and Fetus Associated With MRI Examinations During Pregnancy,” Magnetic Resonance in Medicine 55, no. 4 (2006): 883–893, 10.1002/mrm.20824. 16508913

[5] Krishnamurthy U, Neelavalli J, Mody S, , “MR Imaging of the Fetal Brain at 1.5T and 3.0T Field Strengths: Comparing Specific Absorption Rate (SAR) and Image Quality,” Journal of Perinatal Medicine 43, no. 2 (2015): 209–220, 10.1515/jpm-2014-0268. 25324440 PMC5987203

[6] Barrera CA, Francavilla ML, Serai SD, , “Specific Absorption Rate and Specific Energy Dose: Comparison of 1.5-T Versus 3.0-T Fetal MRI,” Radiology 295, no. 3 (2020): 664–674, 10.1148/radiol.2020191550. 32255418

[7] Abaci Turk E, Yetisir F, Adalsteinsson E, , “Individual Variation in Simulated Fetal SAR Assessed in Multiple Body Models,” Magnetic Resonance in Medicine 83, no. 4 (2020): 1418–1428, 10.1002/mrm.28006. 31626373 PMC6949376

[8] Yetisir F, Abaci Turk E, Guerin B, , “Safety and Imaging Performance of Two-Channel RF Shimming for Fetal MRI at 3T,” Magnetic Resonance in Medicine 86, no. 5 (2021): 2810–2821, 10.1002/mrm.28895. 34240759 PMC8530882

[9] Yetisir F, Abaci Turk E, Adalsteinsson E, Wald LL, and Grant PE, “Local SAR Management Strategies to Use Two-Channel RF Shimming for Fetal MRI at 3 T,” Magnetic Resonance in Medicine 91, no. 3 (2024): 1165–1178, 10.1002/mrm.29913. 37929768 PMC10843691

[10] Hennig J, Weigel M, and Scheffler K, “Multiecho Sequences With Variable Refocusing Flip Angles: Optimization of Signal Behavior Using Smooth Transitions Between Pseudo Steady States (TRAPS),” Magnetic Resonance in Medicine 49, no. 3 (2003): 527–535, 10.1002/mrm.10391. 12594756

[11] Weigel M and Hennig J, “Development and Optimization of Weighted Methods With Reduced RF Power Deposition (Hyperecho-TSE) for Magnetic Resonance Imaging,” Zeitschrift für Medizinische Physik 18, no. 3 (2008): 151–161, 10.1016/j.zemedi.2008.01.008. 18826158

[12] Busse RF, Hariharan H, Vu A, and Brittain JH, “Fast Spin Echo Sequences With Very Long Echo Trains: Design of Variable Refocusing Flip Angle Schedules and Generation of Clinical T2 Contrast,” Magnetic Resonance in Medicine 55, no. 5 (2006): 1030–1037, 10.1002/mrm.20863. 16598719

[13] Busse RF, Brau ACS, Vu A, , “Effects of Refocusing Flip Angle Modulation and View Ordering in 3D Fast Spin Echo,” Magnetic Resonance in Medicine 60, no. 3 (2008): 640–649, 10.1002/mrm.21680. 18727082 PMC2760745

[14] Loening AM, Saranathan M, Ruangwattanapaisarn N, Litwiller DV, Shimakawa A, and Vasanawala SS, “Increased Speed and Image Quality in Single-Shot Fast Spin Echo Imaging via Variable Refocusing Flip Angles,” Journal of Magnetic Resonance Imaging 42, no. 6 (2015): 1747–1758, 10.1002/jmri.24941. 26094580 PMC4684814

[15] Herrmann J, Nickel D, Mugler JP, , “Development and Evaluation of Deep Learning-Accelerated Single-Breath-Hold Abdominal HASTE at 3 T Using Variable Refocusing Flip Angles,” Investigative Radiology 56, no. 10 (2021): 645–652, 10.1097/RLI.0000000000000785. 33965966

[16] Keerthivasan MB, Galons JP, Johnson K, , “Abdominal T2-Weighted Imaging and T2 Mapping Using a Variable Flip Angle Radial Turbo Spin-Echo Technique,” Journal of Magnetic Resonance Imaging 55, no. 1 (2022): 289–300, 10.1002/jmri.27825. 34254382 PMC8678192

[17] Zhao L, Chang C, and Alsop DC, “Controlling T2 Blurring in 3D Rare Arterial Spin Labeling Acquisition Through Optimal Combination of Variable Flip Angles and k-Space Filtering,” Magnetic Resonance in Medicine 80, no. 4 (2018): 1391–1401, 10.1002/mrm.27118. 29427325 PMC6085152

[18] Keerthivasan MB, Winegar B, Udayasankar U, Bilgin A, Altbach M, and Saranathan M, “An Optimized Single-Shot Sequence for Fast T2w Imaging of the Brain,” in Proceedings of the Joint Annual Meeting ISMRM-ESMRMB (2018), 0316.

[19] Arefeen Y, Gagoski B, Turk E, , “Single-Shot T2-Weighted Fetal MRI With Variable Flip Angles, Full K-Space Sampling, and Nonlinear Inversion: Towards Improved SAR and Sharpness,” in Proceedings of the 2020 ISMRM & SMRT Virtual Conference and Exhibition (John Wiley and Sons Inc, 2020), 2574.

[20] Arefeen Y, Kim TH, Halder J, , “Rapid Fetal HASTE Imaging Using Variable Flip Angles and Simultaneous Multislice Wave-LORAKS,” in Proceedings of the 2021 ISMRM Annual Meeting and Exhibition (2021), 0347.

[21] Arefeen Y, Gagoski B, Bilgic B, Grant E, and Adelsteinsson E, “Improved Acquisition Efficiency in T2-Weighted Fetal MRI With Optimized Variable Flip Angles and Prospective Wave-Encoding,” in Proceedings of the Joint Annual Meeting ISMRM-ESMRMB 2022 & ISMRT Annual Meeting (2022), 0740.

[22] Wu J, Qin F, Tian F, , “Age-Specific Optimization of the T2-Weighted MRI Contrast in Infant and Toddler Brain,” Magnetic Resonance in Medicine 93, no. 3 (2025): 1014–1025, 10.1002/mrm.30339. 39428905

[23] Lajous H, Roy CW, Hilbert T, , “A Fetal Brain Magnetic Resonance Acquisition Numerical Phantom (FaBiAN),” Scientific Reports 12, no. 1 (2022): 8682, 10.1038/s41598-022-10335-4. 35606398 PMC9127105

[24] Wales D and Doye J, “Global Optimization by Basin-Hopping and the Lowest Energy Structures of Lennard-Jones Clusters Containing up to 110 Atoms,” Journal of Physical Chemistry A 101, no. 28 (1997): 5111–5116, 10.1021/jp970984n.

[25] Griswold MA, Jakob PM, Heidemann RM, , “Generalized Autocalibrating Partially Parallel Acquisitions (GRAPPA),” Magnetic Resonance in Medicine 47, no. 6 (2002): 1202–1210, 10.1002/mrm.10171. 12111967

[26] Weigel M, “Extended Phase Graphs: Dephasing, RF Pulses, and Echoes—Pure and Simple: Extended Phase Graphs,” Journal of Magnetic Resonance Imaging 41, no. 2 (2015): 266–295, 10.1002/jmri.24619. 24737382

[27] Stupic KF, Ainslie M, Boss MA, , “A Standard System Phantom for Magnetic Resonance Imaging,” Magnetic Resonance in Medicine 86, no. 3 (2021): 1194–1211, 10.1002/mrm.28779. 33847012 PMC8252537

[28] Reeder SB, Wintersperger BJ, Dietrich O, , “Practical Approaches to the Evaluation of Signal-to-Noise Ratio Performance With Parallel Imaging: Application With Cardiac Imaging and a 32-Channel Cardiac Coil,” Magnetic Resonance in Medicine 54, no. 3 (2005): 748–754, 10.1002/mrm.20636. 16088885

[29] Dietrich O, Raya JG, Reeder SB, Reiser MF, and Schoenberg SO, “Measurement of Signal-to-Noise Ratios in MR Images: Influence of Multichannel Coils, Parallel Imaging, and Reconstruction Filters,” Journal of Magnetic Resonance Imaging 26, no. 2 (2007): 375–385, 10.1002/jmri.20969. 17622966

[30] Gudbjartsson H and Patz S, “The Rician Distribution of Noisy MRI Data,” Magnetic Resonance in Medicine 34, no. 6 (1995): 910–914, 10.1002/mrm.1910340618. 8598820 PMC2254141

[31] Gholipour A, Estroff JA, and Warfield SK, “Robust Super-Resolution Volume Reconstruction From Slice Acquisitions: Application to Fetal Brain MRI,” IEEE Transactions on Medical Imaging 29, no. 10 (2010): 1739–1758, 10.1109/TMI.2010.2051680. 20529730 PMC3694441

[32] Kuklisova-Murgasova M, Quaghebeur G, Rutherford MA, Hajnal JV, and Schnabel JA, “Reconstruction of Fetal Brain MRI With Intensity Matching and Complete Outlier Removal,” Medical Image Analysis 16, no. 8 (2012): 1550–1564, 10.1016/j.media.2012.07.004. 22939612 PMC4067058

[33] Ebner M, Wang G, Li W, , “An Automated Framework for Localization, Segmentation and Super-Resolution Reconstruction of Fetal Brain MRI,” NeuroImage 206 (2020): 116324, 10.1016/j.neuroimage.2019.116324. 31704293 PMC7103783

[34] Weigel M, Helms G, and Hennig J, “Investigation and Modeling of Magnetization Transfer Effects in Two-Dimensional Multislice Turbo Spin Echo Sequences With Low Constant or Variable Flip Angles at 3 T,” Magnetic Resonance in Medicine 63, no. 1 (2010): 230–234, 10.1002/mrm.22145. 19859950

[35] Gagoski B, Xu J, Wighton P, , “Automated Detection and Re-acquisition of Motion-Degraded Images in Fetal HASTE Imaging at 3 T,” Magnetic Resonance in Medicine 87, no. 4 (2022): 1914–1922, 10.1002/mrm.29106. 34888942 PMC8810713

[36] Salehi SSM, Khan S, Erdogmus D, and Gholipour A, “Real-Time Deep Pose Estimation With Geodesic Loss for Image-to-Template Rigid Registration,” IEEE Transactions on Medical Imaging 38, no. 2 (2019): 470–481, 10.1109/TMI.2018.2866442. 30138909 PMC6438698

[37] Neves Silva S, McElroy S, Aviles Verdera J, , “Fully Automated Planning for Anatomical Fetal Brain MRI on 0.55T,” Magnetic Resonance in Medicine 92, no. 3 (2024): 1263–1276, 10.1002/mrm.30122. 38650351

[38] Singh A, Salehi SSM, and Gholipour A, “Deep Predictive Motion Tracking in Magnetic Resonance Imaging: Application to Fetal Imaging,” IEEE Transactions on Medical Imaging 39, no. 11 (2020): 3523–3534, 10.1109/TMI.2020.2998600. 32746102 PMC7787194

