# Supplementary material for: Variable Flip Angle Optimization for Fetal Brain Imaging With Reduced Specific Absorption Rate
Source: NMR Biomed. Author manuscript; Available in PMC 2026 Jul 1. (PMC13156923; doi:10.1002/nbm.70075)
Supplement: Supplementary Material [file NIHMS2167014-supplement-Supplementary_Material.docx]

**Supporting Information (Tables S1 and S2, and Figures S1-S7):**

**Supporting information Table S1.** Image quality grading scale

| Score | Diagnostic value | Description | Clinical value |
| --- | --- | --- | --- |
| 1 | Uninterpretable examination | The examination cannot be interpreted due to severe artifacts or technical issues. | Non-diagnostic |
| 2 | Poor image quality | Significant artifacts or deficiencies present. Limited anatomical details are visible, making the examination marginally usable for diagnostic purposes. | Non-diagnostic |
| 3 | Acceptable image quality | Moderate artifacts or image irregularities present but do not prevent the identification of major anatomical structures. Suitable for basic diagnostic purposes and some biometric measurements. | Partially Diagnostic |
| 4 | Good image quality | Minor artifacts present that do not interfere with the interpretation. Most anatomical details are clear and discernible. Suitable for comprehensive diagnostic evaluation and biometric analysis. | Diagnostic |
| 5 | Excellent image quality | Minimal to no artifacts. High-resolution images with clear visibility of anatomical structures. Ideal for detailed diagnostic evaluation and precise biometric measurements. | Diagnostic |

**Supporting Information Table S2.** Image metrics calculated from estimated signal evolutions for the CFA and proposed VFA scans, assuming ISMRM/NIST system phantom relaxation values

| Sequence | SAR (au) | Relative contrast (au) | Tissue | AUDC (au) | Signal (au) | FWHM (pixels) | Motion sensitivity (%) |
| --- | --- | --- | --- | --- | --- | --- | --- |
| CFA | 1 (1.49×10^6^) | 1 (0.162) | GM | 1 (0.182) | 1 (0.582) | 1 (2.625) | 1 (0.997) |
|  |  |  | WM | 1 (0.258) | 1 (0.694) | 1 (1.875) | 1 (0.997) |
| VFA | 0.637 (9.52×10^5^) | 0.989 (0.16) | GM | 0.994 (0.181) | 1.011 (0.588) | 0.952 (2.5) | 0.996 (0.993) |
|  |  |  | WM | 0.956 (0.247) | 1.009 (0.701) | 1 (1.875) | 0.996 (0.993) |
| Note – The image metrics calculated in Table 1 were re-calculated assuming T_1_ and T_2_ values of the ISMRM/NIST system vials that were selected to best approximate fetal gray and white matter. The metrics with the greatest difference from Table 1 are AUDC (VFA, WM) and FWHM (VFA, GM). AUDC: area under the signal decay curve, FMWH: full-width-at-half-maximum, GM: gray matter, SAR: specific absorption rate, WM: white matter, VFA: variable flip angle, CFA: constant flip angle. | | | | | | | |

| 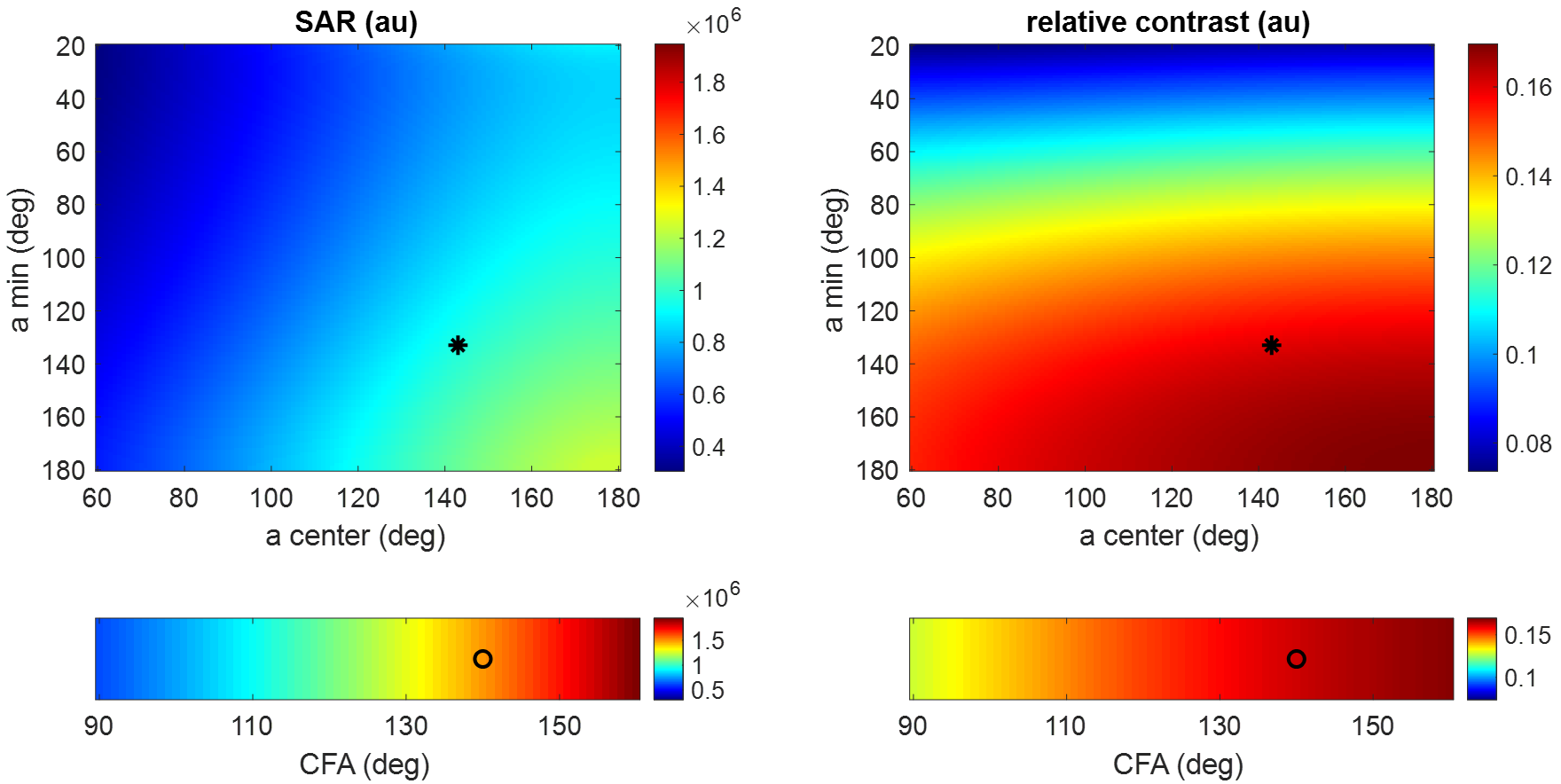 | |
| --- | --- |
| (a) | (b) |
| **Supporting Information Figure S1.** Heat maps displaying the results of (a) SAR and (b) relative GM/WM contrast values (based on simulations) in a grid search over α_min_ and α_cent_ while keeping α_start_ and α_end_ fixed at the values determined during the VFA optimization (α_start_ = 159°, α_end_ = 42°).  The black asterisks show the proposed values (α_min_ = 133°, α_cent_ = 143°) for a trade-off to achieve low SAR and high contrast. Also shown for comparison in the second row are results for the CFA scan with different constant flip angles while all other parameters are held constant. The black circles show the value used in our protocol (140°). As can be seen in the CFA plots, the relative contrast and SAR both drop significantly with lower flip angles. The optimized VFA allowed lowering SAR while maintaining relatively high contrast between GM and WM in fetal brain imaging. SAR: specific absorption rate, VFA: variable flip angle, CFA: constant flip angle. | |

| 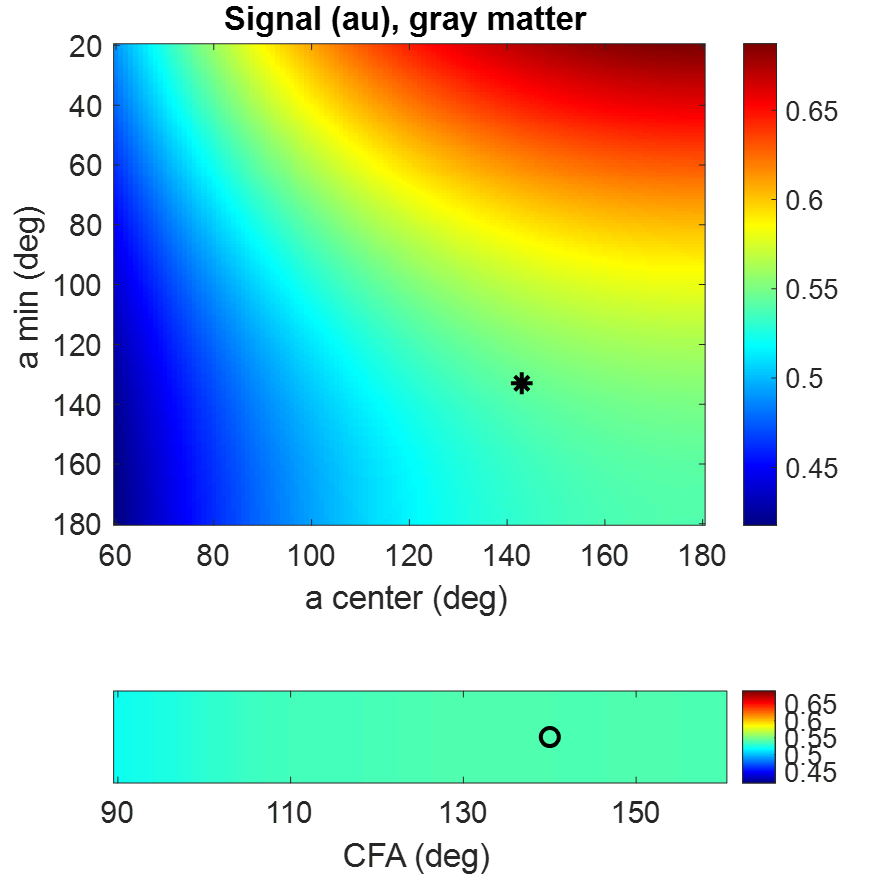 | 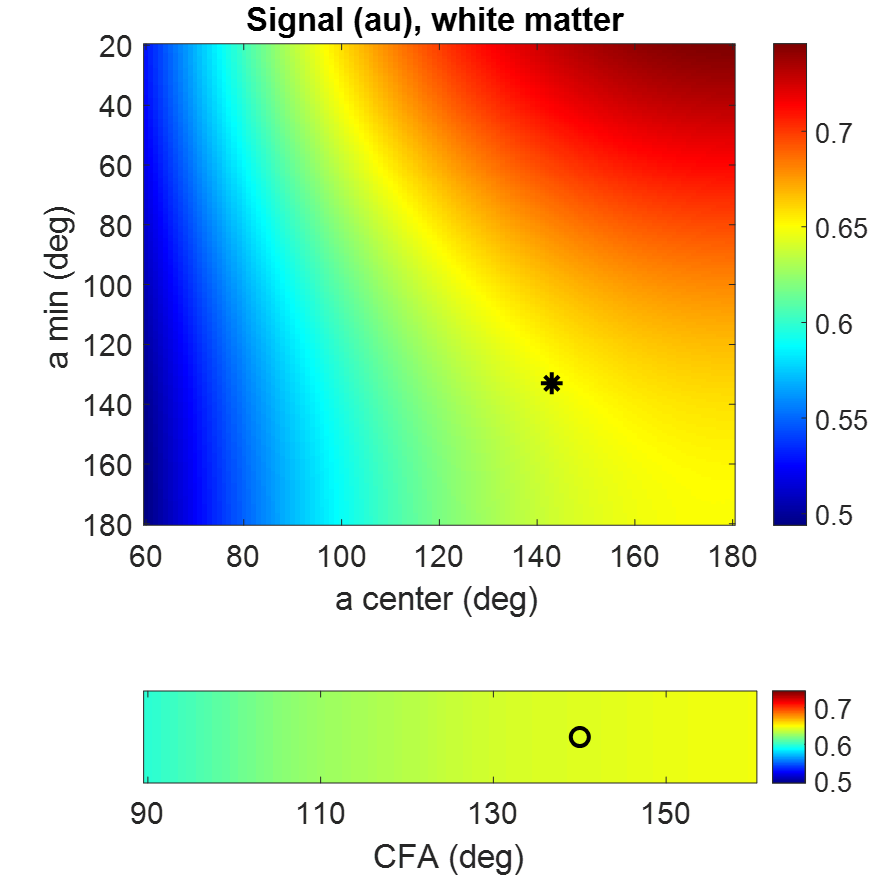 |
| --- | --- |
| (a) | (b) |
| **Supporting Information Figure S2.** Heat maps displaying changes in signal values for the fetal brain GM and WM (based on simulations) in a grid search over α_min_ and α_cent_ while keeping α_start_ and α_end_ fixed at the values determined during the VFA optimization (α_start_ = 159°, α_end_ = 42°).  The black asterisks show the proposed values (α_min_ = 133°, α_cent_ = 143°). Also shown for comparison in the second row are results for the CFA scan with different constant flip angles while all other parameters are held constant. The black circles show the flip angle value used in our protocol (140°). As can be seen, the WM and GM signals drop with higher α_min_ and lower α_cent_. Compared to the CFA scans at the operating constant flip angle, the optimized VFA maintained both WM and GM signal while lowering the SAR. SAR: specific absorption rate, GM: gray matter, WM: white matter, VFA: variable flip angle, CFA: constant flip angle. | |

| 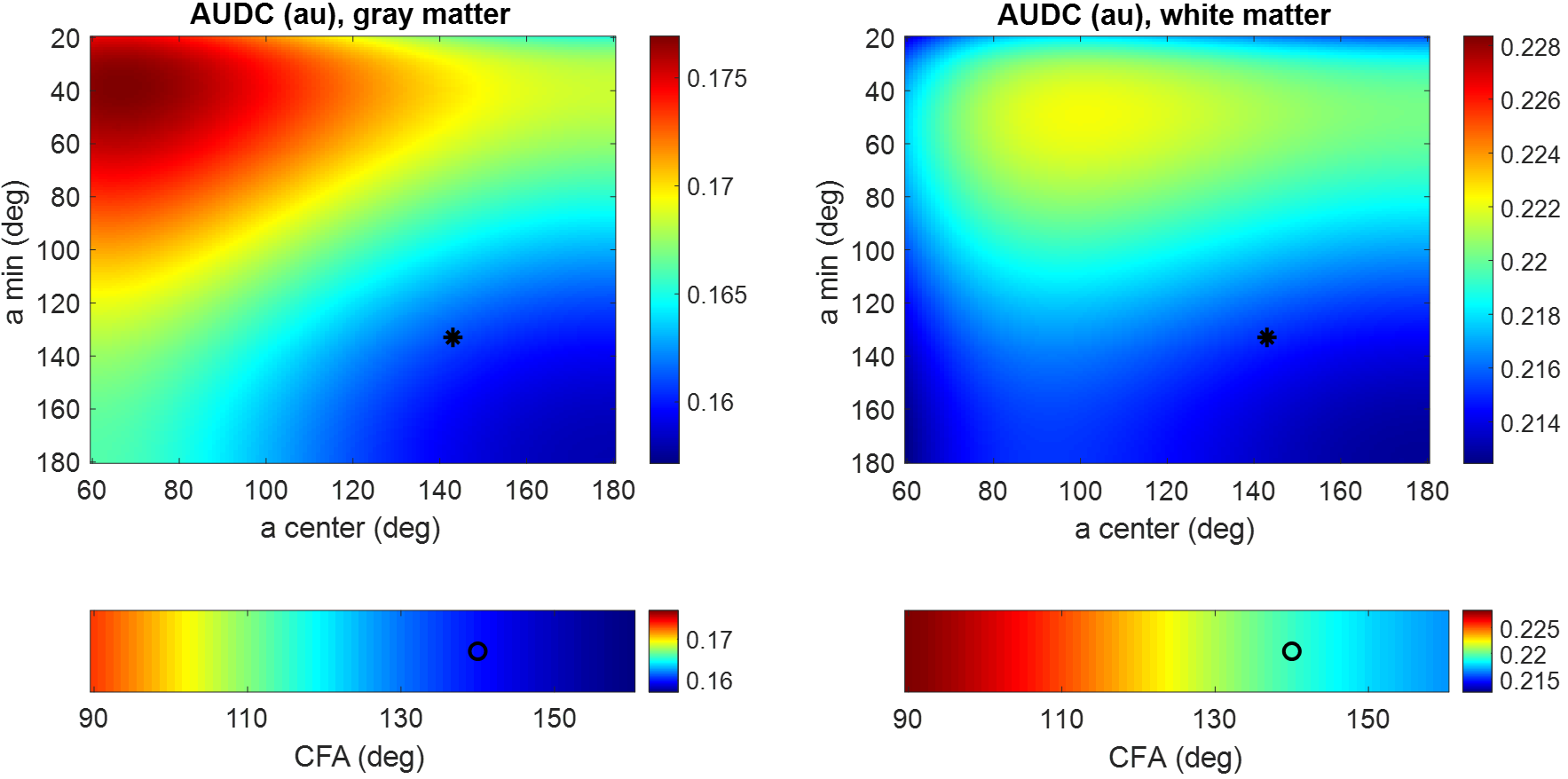 | |
| --- | --- |
| (a) | (b) |
| **Supporting Figure S3.** Heat maps displaying the Area Under the signal Decay Curve (AUDC) of the fetal brain GM and WM (based on simulations) in a grid search over α_min_ and α_cent_ while keeping α_start_ and α_end_ fixed at the values determined during the VFA optimization (α_start_ = 159°, α_end_ = 42°).  The black asterisks show the proposed values (α_min_ = 133°, α_cent_ = 143°). Also shown for comparison in the second row are AUDC values for the CFA scan with different constant flip angles while all other parameters are held constant. The black circles show the value used in our CFA protocol (140°). Simulations show that the AUDC values drop with higher flip angles, and the optimized VFA maintains similar AUDC as the CFA scan while reducing SAR. SAR: specific absorption rate, GM: gray matter, WM: white matter, VFA: variable flip angle, CFA: constant flip angle. | |

| 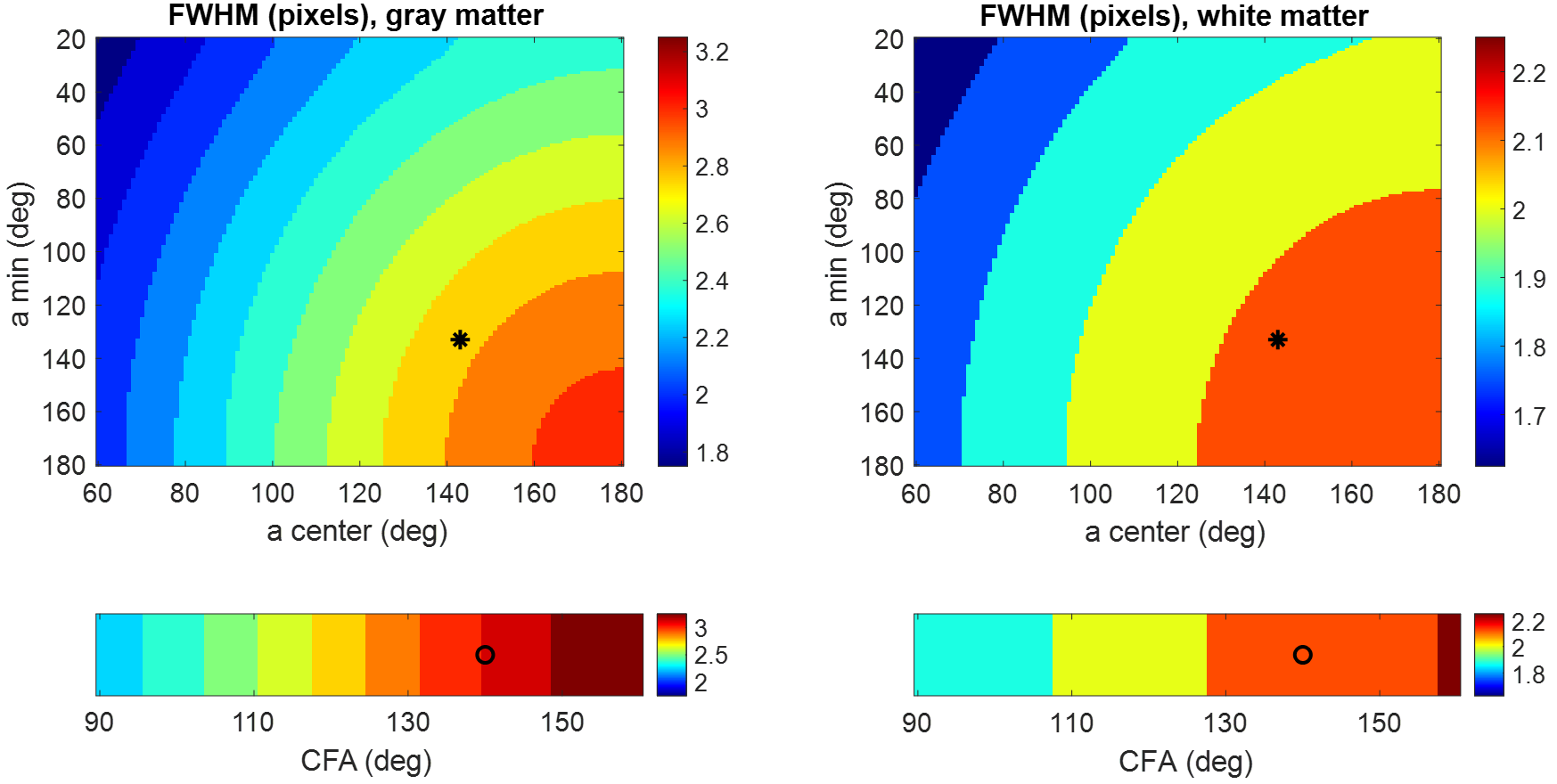 | |
| --- | --- |
| (a) | (b) |
| **Supporting Information Figure S4.** Heat maps displaying the changes (based on simulations) in Full Width at Half Maximum (FWHM) of the point spread function of the GM (a) and WM (b) as a proxy for spatial resolution, in a grid search over α_min_ and α_cent_ while keeping α_start_ and α_end_ fixed at the values determined during the VFA optimization (α_start_ = 159°, α_end_ = 42°).  The black asterisks show the proposed values (α_min_ = 133°, α_cent_ = 143°). Also shown for comparison in the second row are the FWHM values for the CFA scan with different constant flip angles while all other parameters are held constant. The black circles show the value used in our CFA protocol (140°). Simulations indicate that the FWHM values may slightly decrease with the VFA compared to the CFA protocol. GM: gray matter, WM: white matter, VFA: variable flip angle, CFA: constant flip angle. | |

| 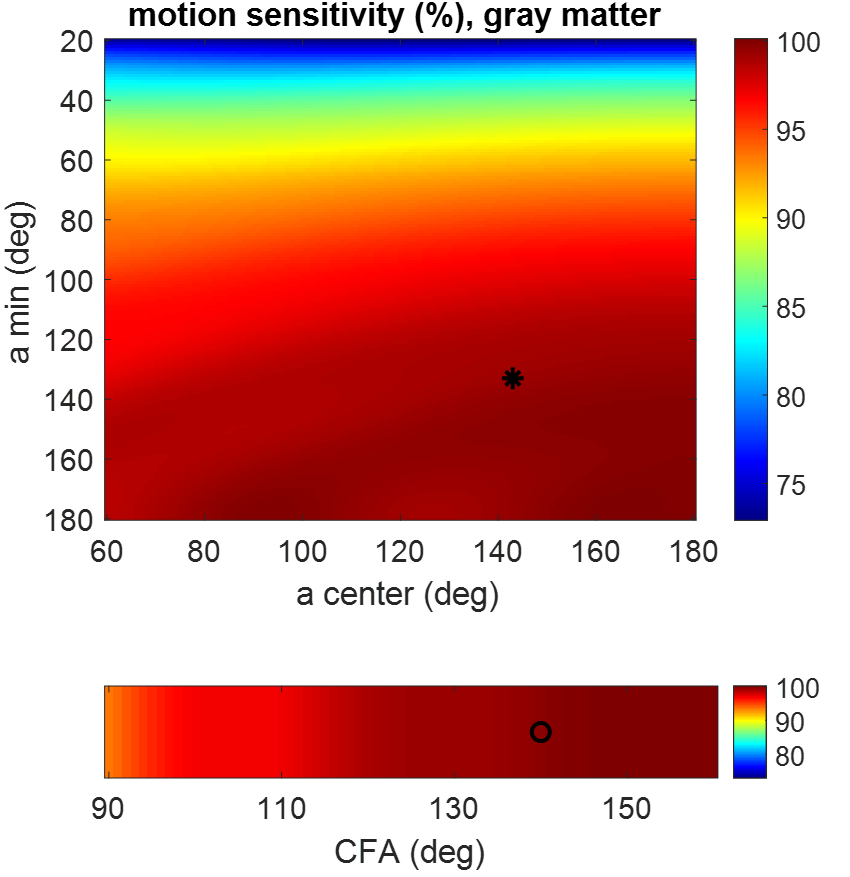 | 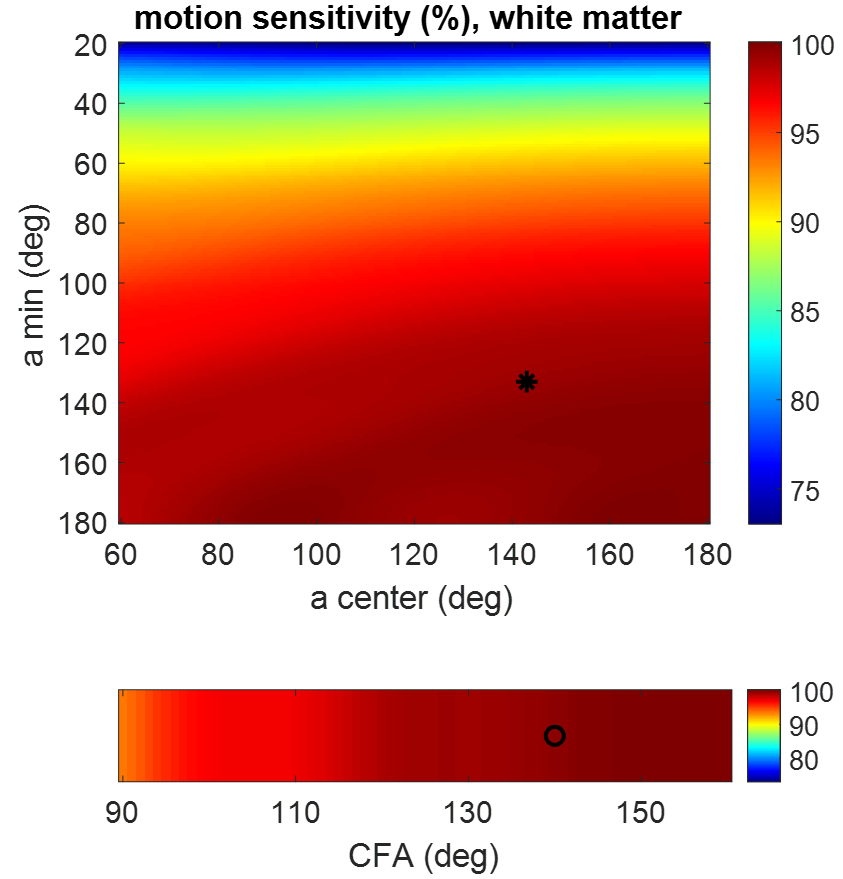 |
| --- | --- |
| (a) | (b) |
| **Supporting Information Figure S5.** Heat maps displaying simulated changes in motion sensitivity of the GM (a) and WM (b) signals in a grid search over α_min_ and α_cent_ while keeping α_start_ and α_end_ fixed at the values determined during the VFA optimization (α_start_ = 159°, α_end_ = 42°).  The black asterisks show the proposed values (α_min_ = 133°, α_cent_ = 143°). Also shown for comparison in the second row are results for the CFA scan with different constant flip angles while all other parameters are held constant. The black circles show the value used in our protocol (140°). The units of motion sensitivity are % of the signal without motion. These simulation results indicate that the VFA signals are only slightly more sensitive to motion than the CFA signals. GM: gray matter, WM: white matter, VFA: variable flip angle, CFA: constant flip angle. | |


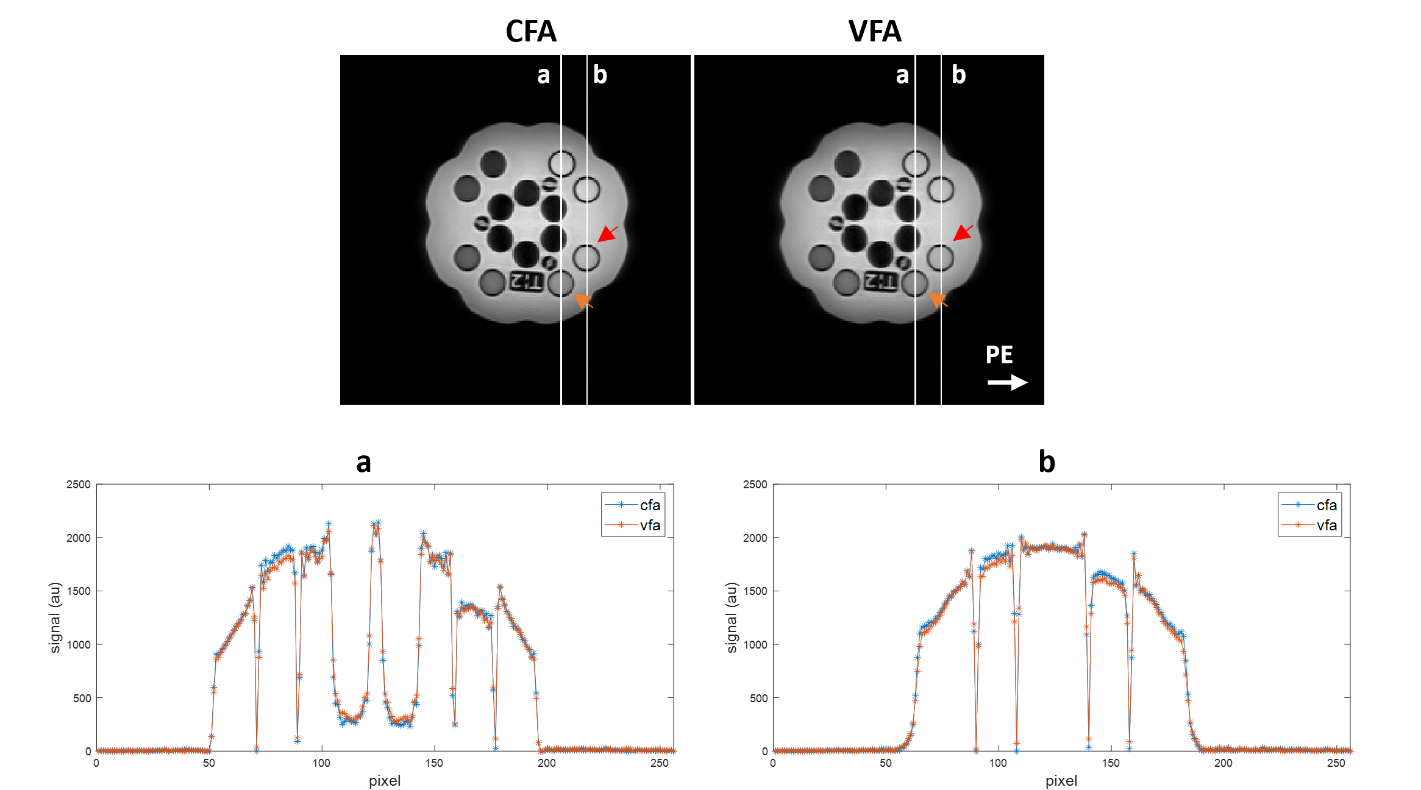


**Supporting Information Figure S6.** Line profiles in the readout direction across the T_2_ layer of the ISMRM/NIST standard phantom imaged with the CFA scan and the proposed VFA scan. The line profiles include the vials with T_1_/T_2_ values closest to fetal gray matter (orange arrow) and white matter (red arrow). VFA: variable flip angle, CFA: constant flip angle.


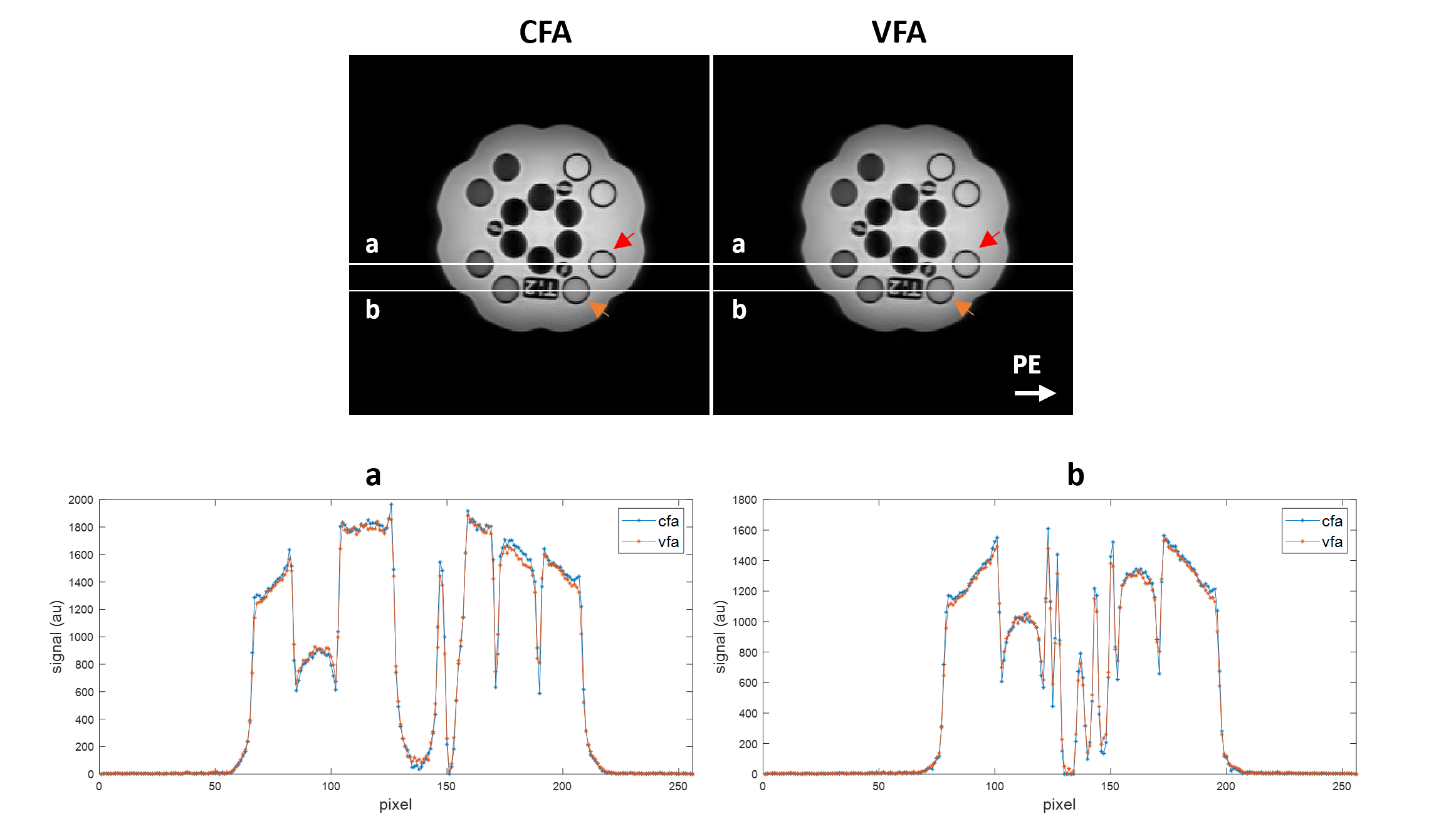


**Supporting Information Figure S7.** Line profiles in the phase encode direction across the T_2_ layer of the ISMRM/NIST standard phantom imaged with the CFA scan and the proposed VFA scan. The line profiles include the vials with T_1_/T_2_ values closest to fetal gray matter (orange arrow) and white matter (red arrow). VFA: variable flip angle, CFA: constant flip angle.
